# Elucidation of the Mechanisms and Effective Substances of Paeoniae Radix Rubra Against Toxic Heat and Blood Stasis Syndrome With a Stage-Oriented Strategy

**DOI:** 10.3389/fphar.2022.842839

**Published:** 2022-03-04

**Authors:** Jing-Jing Xu, Feng Xu, Wei Wang, Yi-Fan Zhang, Bei-Quan Hao, Ming-Ying Shang, Guang-Xue Liu, Yao-Li Li, Shu-Bin Yang, Xuan Wang, Shao-Qing Cai

**Affiliations:** ^1^ State Key Laboratory of Natural and Biomimetic Drugs, School of Pharmaceutical Sciences, Peking University, Beijing, China; ^2^ School of Pharmacy, Heilongjiang University of Chinese Medicine, Harbin, China

**Keywords:** Paeoniae Radix Rubra, effective substance, pharmacodynamic mechanism, stage-oriented strategy, metabonomics, partial least square regression, toxic heat and blood stasis

## Abstract

In the clinical practice of traditional Chinese medicine, toxic heat and blood stasis syndrome (THBSS) is a common syndrome observed in various critical diseases. Paeoniae Radix Rubra (PRR) has known therapeutic effects on THBSS. However, its pharmacodynamic mechanisms and effective substances in the treatment of THBSS still need further elucidation. Our previous study indicated that THBSS had three stages of progression, and the abnormal biochemical indices of each stage were different. Therefore, this study aimed to elucidate the pharmacodynamic mechanisms and effective substances of PRR for the treatment of THBSS with a stage-oriented strategy. Specifically, research was performed separately in two stable stages of THBSS: the excessive heat and little blood stasis (EHLBS) and blood stasis (BS) stages. THBSS model rats, at different time periods after syndrome initiation (first 5 h for EHLBS and 24 h later for BS), were used to conduct the two-stage investigation. Targeted metabonomics analysis was performed to elucidate the pharmacodynamic mechanisms of PRR in the treatment of EHLBS or BS. Based on the relationship between the individual differences in blood drug concentrations and pharmacodynamic effects, partial least squares regression analysis was employed to screen for the effective substances from the original constituents and metabolites of PRR. We found that PRR could upregulate primary bile acid biosynthesis, glycerophospholipid metabolism, ether lipid metabolism, and five amino acid metabolic pathways (e.g., arginine and proline metabolism) to treat EHLBS. Meanwhile, PRR alleviated BS by upregulating primary bile acid biosynthesis and downregulating glycerophospholipid metabolism. But PRR had no obvious effects on ether lipid metabolism and amino acid metabolism in this stage. In total, 17 and 9 potential effective substances were found in the EHLBS and BS stages, respectively, among which there were only five common compounds between the two stages. To our knowledge, sixteen compounds were regarded as potential effective substances of PRR for the first time. Therefore, the pharmacodynamic mechanisms and effective substances of PRR in the treatment of EHLBS and BS were partly different. Overall, this stage-oriented strategy provides a new way to study the pharmacodynamic mechanisms and effective substances of traditional Chinese drugs.

## 1 Introduction

Traditional Chinese drugs (TCDs) have been used in China for thousands of years and have recently received global attention. It is important to select suitable strategies for TCD development to meet the requirements of modern medicine (Li et al., 2008; Zhang B. et al., 2019). Elucidation of the pharmacodynamic mechanisms and the effective substances of TCDs is an important aspect of modern TCD research. In the past few decades, various strategies based on multiple perspectives have been utilized, including chinmedomics (Zhang A.-H. et al., 2019), systems pharmacology (Zhang W. J. et al., 2019), and metabonomics (Wang et al., 2017). Since the basic theory of traditional Chinese medicine (TCM) is the essential basis of TCD applications, how to carry out modern TCD research under the guidance of TCM basic theory is still a critical issue.

Syndrome differentiation and treatment, one of the core concepts of TCM basic theory, refers to the use of an appropriate TCD according to the corresponding syndrome. Thus, the existence of the corresponding syndrome is considered a prerequisite of TCD research (Zhang A.-H. et al., 2019). As progression is a critical characteristic of a syndrome, it is common practice to choose different treatments or TCDs for different stages of the syndrome (Wang and Zhang 2009). Thus, both the existence and stage of the syndrome should be considered. Overall, we believe that a research strategy based on the specific stages of a TCD’s corresponding syndrome (we named this “stage-oriented strategy”) that conforms to TCM basic theory is suitable for modern research.

Paeoniae Radix Rubra (PRR) is derived from the dried roots of *Paeonia lactiflora* Pall. or *Paeonia veitchii* Lynch. PRR is an important TCD used to cure toxic heat and blood stasis syndrome (THBSS), which is a severe syndrome observed in various acute and critical diseases, such as coronary heart disease (Xu et al., 2019), myocardial infarction (Huang 2000), ischemic stroke (Zhang C. et al., 2019), and multiple organ failure syndrome (Xie 2013). Accumulated evidence from various studies suggests that PRR has definite therapeutic effects on THBSS. PRR can alleviate the symptoms of THBSS in animal models (Xie et al., 2005). Additionally, Ke-Ji Chen, a Chinese academician, regards PRR as the main TCD in the clinical treatment of coronary heart disease belonging to THBSS (Zhang and Xie 2008). Several studies have been conducted on the phytochemistry (Yang et al., 2020), pharmacology (Ma et al., 2018), and metabolism (Liang et al., 2013) of PRR. However, its pharmacodynamic mechanisms and related effective substances in the treatment of THBSS still need further elucidation.

According to the TCM basic theory, the pathogenesis of THBSS is that heat-toxin invades the body, then consumes body fluid, and thickens the blood, finally leads to blood stasis. The related modern researches elucidate this process as that inflammation induces abnormal coagulation and hemorheology, and microcirculation disorder, finally leads to ischemia, hypoxia, and blood stasis of tissues and organs (Yang et al., 2010). Meanwhile, inflammatory cytokines (IL-6, TNF-α, etc.), hemorheological indexes (whole blood viscosity, plasma viscosity), temperature, and coagulation time (PT, TT, APTT, etc.) are regarded as the common evaluation indexes of THBSS in clinical and nonclinical researches (Wang and Xu 2009). Our previous research systematically investigated these evaluation indexes, pathological indices, and endogenous metabolites of lipopolysaccharide (LPS)/carrageenan (CAR)-induced THBSS model rats (a well-established animal model with very similar pathogenesis and manifestations of THBSS) at different time points (2, 5, 8, 12, 24 h, etc.) after modeling. And we confirmed that THBSS definitely had three stages of progression in model rats, and clarified the time period of each stage: the excessive heat and little blood stasis (EHLBS) stage (2–5 h), stasis heat coexistence (SHC) stage (8–12 h), and blood stasis (BS) stage (after 24 h). Meanwhile, the determination results of physical signs, biochemical and pathological indices of THBSS model rats were different among the three stages. For example, in EHLBS stage, the model rats mainly showed physical signs of heat syndrome (elevated forehead temperature, red paws, etc.), elevated IL-6 and TNF-α, etc., whereas in BS stage, the model rats mainly exhibited physical signs of blood stasis syndrome (thrombi in tails, ecchymosis in ears, etc.), elevated whole blood viscosity, and shortened prothrombin time, etc. (Xu et al., 2017). Based on these results, the present work aimed to elucidate the pharmacodynamic mechanisms and effective substances of PRR for the treatment of THBSS in LPS/CAR-induced THBSS rat model using a stage-oriented strategy. Specifically, researches were performed separately in two stable stages of THBSS: EHLBS and BS stages.

Meanwhile, untargeted metabonomics analysis conducted in our previous study revealed that amino acid metabolism (histidine metabolism, arginine and proline metabolism, etc.) and energy metabolism (citrate cycle, pyruvate metabolism, etc.) were significantly disturbed during THBSS progression, which were closely related to the inflammation and hemorheology and coagulation abnormalities (the main manifestations in model rats during the progression of THBSS) (Xu et al., 2017). It is reported that bile acids are important signal molecules in energy metabolism and can affect coagulation through farnesol receptor X (Poland and Flynn 2021). Phospholipids play important roles in inflammation, hemorheology, coagulation, and microcirculation (Deguchi et al., 2017). Therefore, to clarify the pharmacodynamic mechanisms of PRR in the treatment of EHLBS or BS, targeted metabonomics analysis was conducted on amino acids, bile acids, and phospholipids. Furthermore, the effective substances of PRR for the treatment of EHLBS or BS were discovered by exploring the relationship between the differences in blood drug concentrations (the peak areas of the original constituents and metabolites) and the resulting differences in pharmacodynamic effects using partial least squares regression (PLSR) (Gegentana et al., 2020). To the best of our knowledge, this is the first systematic study on the pharmacodynamic mechanisms and effective substances of PRR in the treatment of THBSS, and the first study to investigate a TCD based on different stages of its corresponding syndrome.

## 2 Materials and Methods

### 2.1 Materials

LPS (Lot No. 095M4164V) and CAR (Lot No. SLBK3896V) were purchased from Sigma-Aldrich (St. Louis, MO, United States). High-performance liquid chromatography (HPLC)-grade methanol and acetonitrile were purchased from Merck Group (Darmstadt, Germany), and HPLC-grade formic acid was purchased from Fisher Scientific Corporation (Loughborough, United Kingdom). Ultra-high purity water (18.2 MΩ, total organic carbon <5 ppb) was prepared using a Millipore Milli-Q Integral 3 Ultrapure Water System (Billerica, MA, United States). Blood coagulation function assay kits were purchased from Sysmex Corporation (Kobe, Japan). Tumor necrosis factor *α* (TNF-α) (Lot No. E-EL-R0019c), and interleukin 6 (IL-6) (Lot No. E-EL-R0015c) assay kits were purchased from Elabscience Biotechnology Co., Ltd. (Wuhan, China). PRR was purchased from Beijing Tianheng Pharmacy (Beijing, China; Lot No. 140701) on 20 May 2015. It was authenticated as dried roots of *P. lactiflora* Pall. by Dr. Feng Xu (School of Pharmaceutical Sciences, Peking University). A voucher sample (No. 7838) was deposited in the Herbarium of Pharmacognosy, School of Pharmaceutical Sciences, Peking University.

### 2.2 Preparation of Paeoniae Radix Rubra Extract

Freeze-dried powder of PRR extract (200.01 g) was prepared from 600 g of dried roots of *P. lactiflora* Pall. using a previously described method (Liang et al., 2013). The extraction ratio is 33.33%.

The HPLC fingerprint of the PRR extract is presented in Supplementary Information S1. The contents of five constituents (paeoniflorin, catechin, oxypaeoniflorin, benzoylpaeoniflorin, and gallic acid) in the PRR extract were also determined (Supplementary Information S2).

### 2.3 Animals

Sixty male Sprague-Dawley rats (180–200 g) were purchased from the Experimental Animal Center of Peking University Health Science Center (Beijing, China) on 25 February 2016, and maintained for 1 week at standard temperature (23 ± 2°C) and humidity (60 ± 5%) in a controlled room with a light/dark cycle of 12 h/12 h. During this period, the animals were allowed free access to food and water. Before each experiment, the rats were fasted for 12 h with free access to water.

Thirty rats were used to investigate the pharmacodynamic mechanisms and effective substances of PRR in the treatment of EHLBS. The rats were randomly divided into three groups: control group A, EHLBS group, and PRR-treated group A (EHLBS + PRR extract). The remaining 30 rats were used to elucidate the pharmacodynamic effects and identify the effective substances of PRR in the treatment of BS. They were randomly divided into the following three groups: control group B, BS group, and PRR-treated group B (BS + PRR extract).

All procedures and care of the rats were performed in accordance with the Guide for Care and Use of Laboratory Animals published by the US National Institutes of Health (revised 2010). The experiments were approved by the Biomedical Ethical Committee of Peking University (approval no. LA 2015–007).

### 2.4 Determination of Dosage Regimen

AS THBSS is a TCM syndrome, there was no relevant western medicine for this syndrome that could be selected as a positive control drug in this study. Before the start of the formal experiment, a pre-experiment was performed. At the beginning of pre-experiment, we took 56 g crude drug (the usual dose in clinic of PRR for the treatment of THBSS) and 280 g crude drug (five-fold the usual dose and close to the highest dose in clinic of PRR for the treatment of THBSS) as reference. And then, the daily doses of PRR in rats were calculated to be 5.04 g crude drug/kg and 25.20 g crude drug/kg by a correction factor equal to the human–rat body surface area ratio (6.3) (Xu et al., 2002). Considering the extraction ratio of PRR in this study was 33.33%, the doses in pre-experiment were set at 1.68 g extract/kg and 8.4 g extract/kg in rats.

Since in the pre-experiment the pharmacodynamic effects of PRR were proportional to its dosages, and the obvious pharmacodynamic effects of PRR were observed at the dose of 1.68 g extract/kg, we finally adopted 1.68 g extract/kg in the formal experiment.

### 2.5 Animal Treatments

**FIGURE 1:**
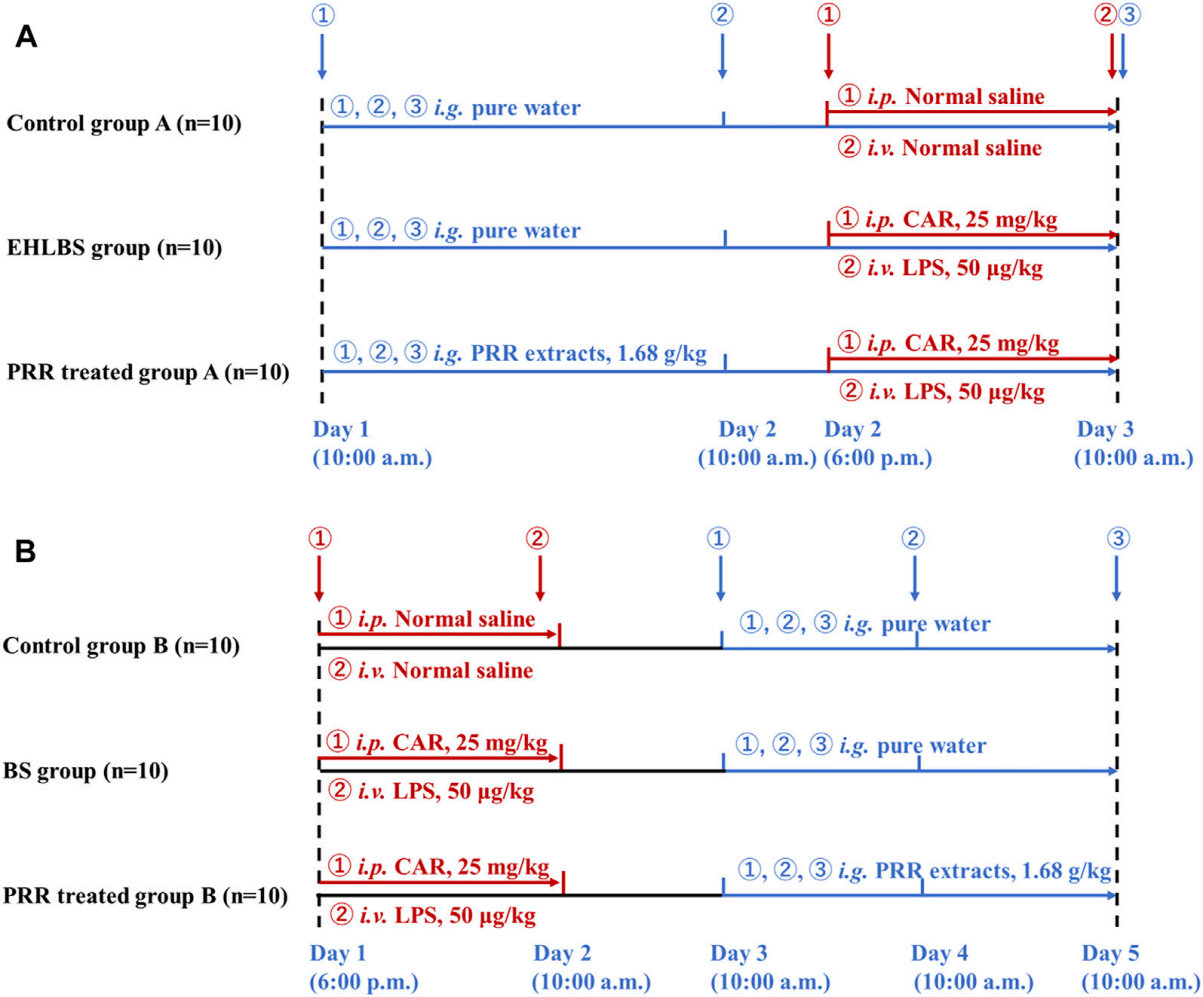
The dosage regimens for EHLBS stage **(A)** and BS stage **(B)**.

Figure 1 shows the detailed dosage regimens in the EHLBS and BS stages. The THBSS rat model used in this study was established using the method described by Liang et al. (2005). Briefly, the rats were first intraperitoneally injected with CAR (25 mg/kg). After 16 h, the rats were administered an intravenous (*i.v.*) injection of LPS (50 μg/kg). The time point of *i. v.* LPS administration was 0 h. In our previous study, we found that during 2–5 h, the rats were in the EHLBS stage and reached the BS stage after 24 h (Xu et al., 2017). Therefore, for the EHLBS-related studies, the freeze-dried PRR powder was dissolved in pure water and administered intragastrically (1.68 g/kg, at 10:00 a.m., once daily) to the rats in PRR-treated group A from the first day to the third day. At 6 p.m. on the second day of the experiment, modeling was initiated on rats in PRR-treated group A and the EHLBS group.

To conduct relevant studies in the BS stage, modeling was initiated on PRR-treated group B and the BS group at 6 p.m. on the first day. At 10:00 a.m. on the third day (24 h after *i. v.* LPS), the rats in PRR-treated group B were intragastrically administered PRR extract (1.68 g/kg, once daily) for three consecutive days.

### 2.6 Sample Collections

Two hours after the last administration of PRR extract, the rats in each group were anesthetized and 8 ml of blood from each rat was collected into a 2 ml sodium citrate vacuum blood collection tube (SCVBCT) and three, 2 ml EDTA vacuum blood collection tubes (EVBCTs). The blood sample in one of the EVBCTs was used to determine whole blood viscosity after standing at room temperature (22–25°C) for 30 min. The blood samples in the SCVBCT and the other two EVBCTs were centrifuged at 3,000 rpm for 10 min to obtain plasma and then stored at −80°C until biochemical and LC-MS analysis. In addition, the liver and lung tissues of control group B, the BS group, and PRR-treated group B were dissected for histological examination.

### 2.7 Determination of Biochemical Parameters

The plasma obtained from one EVBCT was used to assay the levels of IL-6 and TNF-α using enzyme-linked immunosorbent assay kits according to the manufacturer’s instructions. Plasma obtained from SCVBCT was used to test thrombin time (TT), prothrombin time (PT), and activated partial thromboplastin time (APTT) using a Sysmex CA-500 Automatic Blood Coagulation Analyzer (Sysmex Corporation, Kobe, Japan). In addition, 800 μL of anticoagulant blood in the EVBCT was used to determine whole blood viscosity with a BT-300 Automated Viscometer (Nanjing Zhilun Electronic Technology Co., Ltd., Nanjing, China). The forehead temperature of rats was measured by forehead thermometer (MC-872, Omron medical devices (Beijing) Co., Ltd., Beijing, China).

### 2.8 Histological Examination

The liver and lung tissues were fixed in 10% (v/v) formalin for at least 24 h. They were then embedded in paraffin wax, sectioned (3 μm thick), mounted on slides, stained with hematoxylin and eosin (H-E), and observed under a light microscope (OLYMPUS CX21, Olympus Corporation, Tokyo, Japan).

### 2.9 Metabonomics Analysis

Targeted analysis of bile acids, phospholipids, and amino acids was performed according to the general and validated procedures of the analysis and testing platform of the Beijing University of Chemical Technology. The details are provided in Supplementary Information S3.

### 2.10 Analysis of the Original Constituents and Metabolites of Paeoniae Radix Rubra

The original constituents of PRR and their metabolites were analyzed using ultraperformance liquid chromatography-tandem mass spectrometry (UPLC-MS/MS). The details are provided in Supplementary Information S4.

### 2.11 Statistical Analysis

Differences between groups were analyzed by Student’s *t*-tests for normally distributed data or Mann-Whitney tests for non-normally distributed data, using SPSS software (version 20.0; SPSS Inc., Chicago, IL, United States). Statistical significance was set at *p* < 0.05. The trends in the change in endogenous metabolites after modeling or drug administration were obtained through fold-change analysis. Principal component analysis (PCA) and partial least squares-discriminant analysis (PLS-DA) were employed to compare the metabolite profiles of different groups using SIMCA-P software (version 14.1, Umetrics Umea, Sweden). The *p* values of the cross-validated analysis of variance (CV-ANOVA) were calculated to assess the reliability of the PLS-DA model by SIMCA-P software. Pathway analysis was performed using Metaboanalyst 4.0 (http://www.metaboanalyst.ca). The PLSR equation was established with the pharmacodynamic results as Y and the peak area of the original constituents and metabolites of PRR as X, using SIMCA-P software (version 14.1, Umetrics). The correlation analysis network between potential effective substances and endogenous metabolites related to the therapeutic effects of PRR in treating EHLBS or BS was plotted based on Pearson correlation coefficients using Cytoscape (http://cytoscape.org).

## 3 Results and Discussion

### 3.1 Pharmacodynamic Effects of Paeoniae Radix Rubra in Treating Excessive Heat and Little Blood Stasis and Blood Stasis

Inflammatory cytokines (IL-6, TNF-α), temperature, hemorheological indexes (whole blood viscosities, plasma viscosities), and coagulation time (PT, TT, APTT, etc.) are common evaluation indexes of THBSS. Among them, inflammatory cytokines and temperature are biochemical indexes to assess heat syndrome, while whole blood viscosities and coagulation time are biochemical indexes to assess blood stasis syndrome (Wang and Zhang 2009). In our prior study, we found that in the EHLBS stage, the biochemical indexes of heat syndrome were significantly abnormal and the biochemical indexes of blood stasis syndrome were slightly abnormal in THBSS model rats. Whereas in the BS stage, THBSS model rats showed significantly abnormal biochemical indexes of blood stasis syndrome (Xu et al., 2017). Therefore, TNF-α, IL-6, forehead temperature, whole blood viscosities, and coagulation time (APTT, PT, and TT) were selected to evaluate the pharmacodynamic effects of PRR in EHLBS stage. Correspondingly, whole blood viscosities and coagulation time (APTT, PT, and TT) were selected to evaluate the pharmacodynamic effects of PRR in BS stage.

**FIGURE 2:**
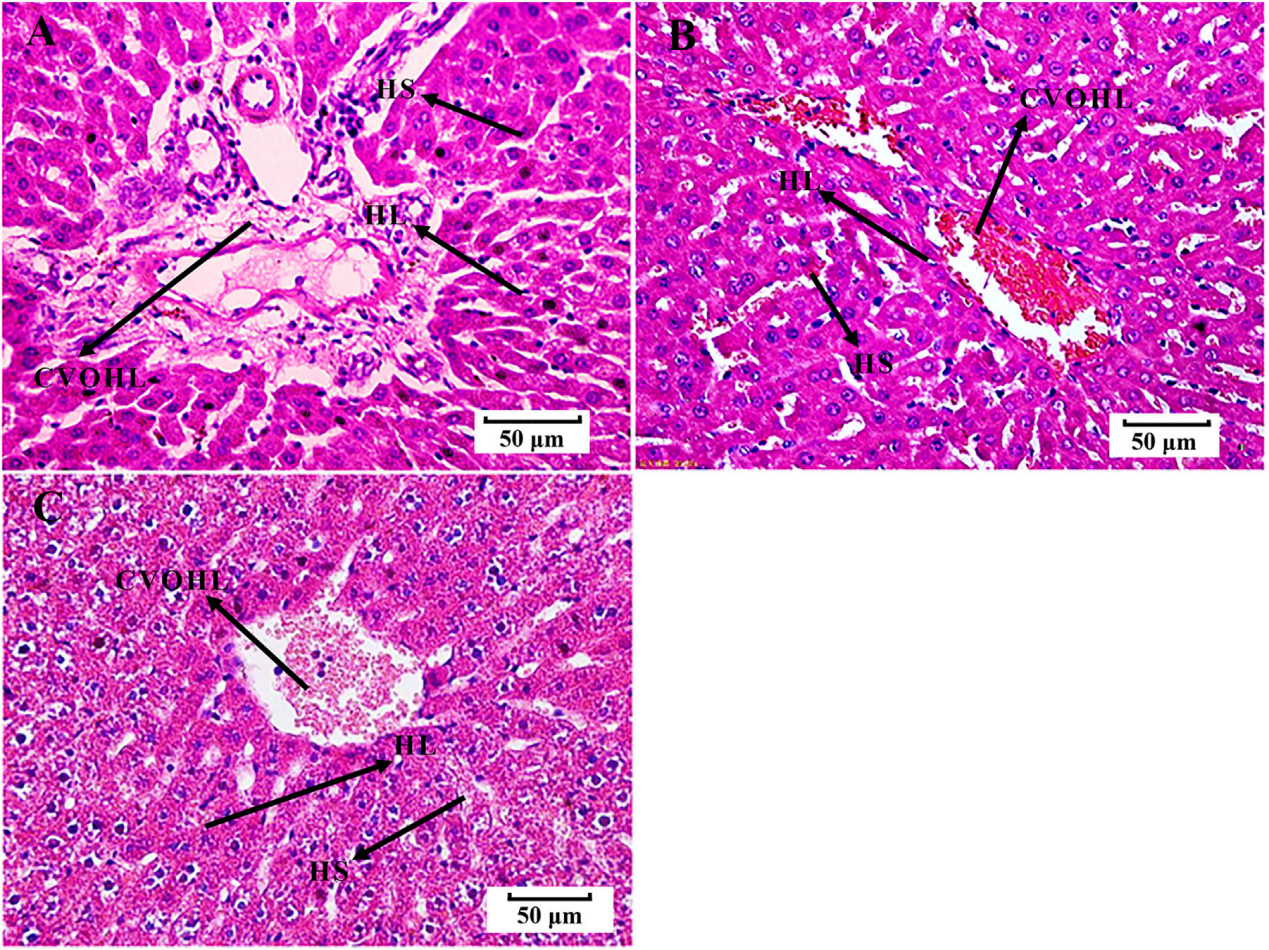
Histological examination of liver tissues (H-E, 50 μm). **(A)** control group B. **(B)** BS group. **(C)** PRR-treated group B. HS, hepatic sinus; CVOHL, central vein of hepatic lobules; HL, hepatic lobules.

**FIGURE 3:**
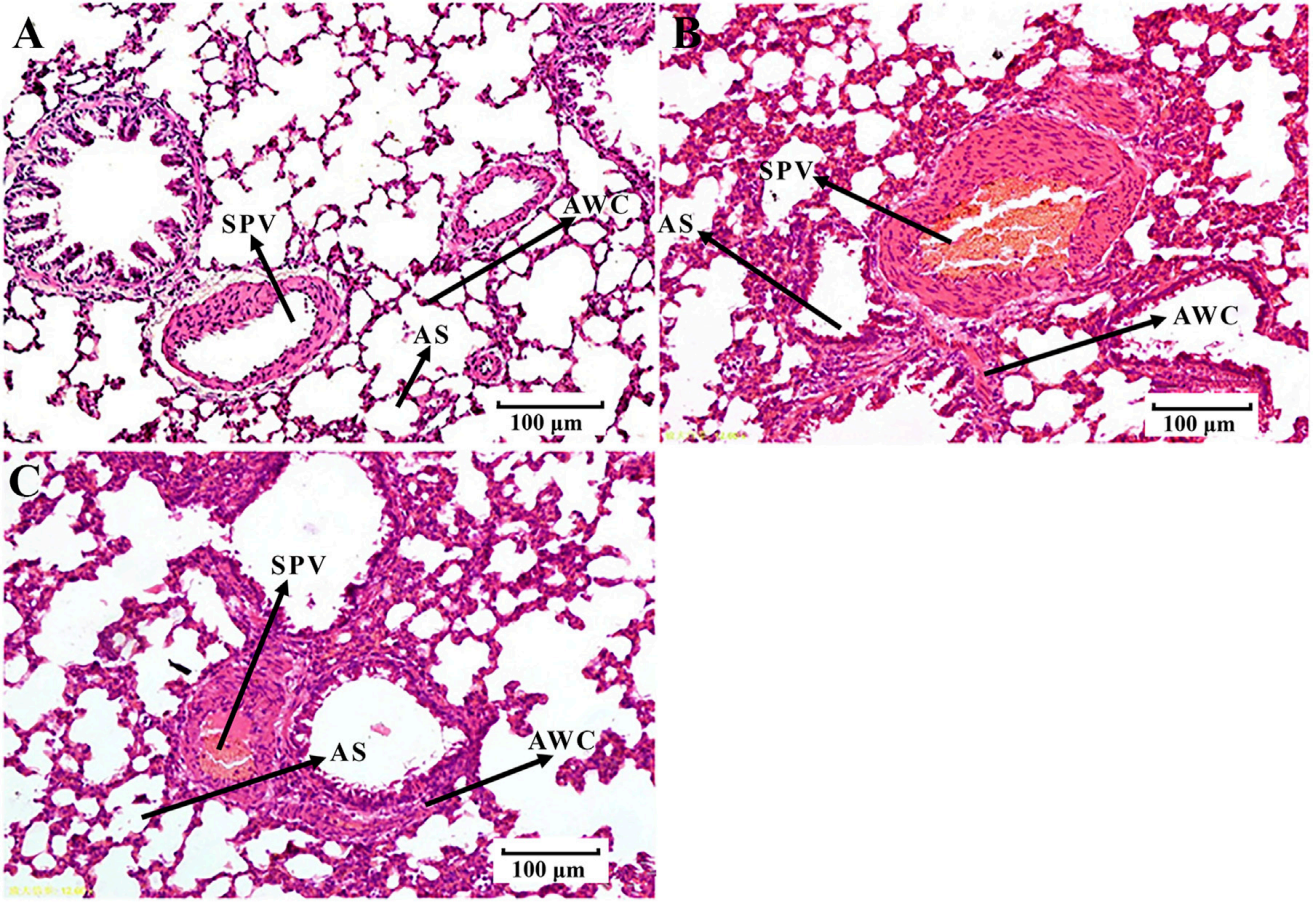
Histological examination of lung tissues (H-E, 100 μm). **(A)** control group B. **(B)** BS group. **(C)** PRR-treated group B. SPV, small pulmonary veins; AWC, alveolar wall capillaries; AS, alveolar space.

We found that the rats in the EHLBS group showed significantly elevated inflammatory cytokine levels (TNF-α and IL-6) (*p* < 0.01), forehead temperatures (*p* < 0.01), whole blood viscosities at all shear stresses (*p* < 0.05), and prolonged APTT (*p* < 0.05), but no significant changes in PT and TT, when compared with the corresponding values of the rats in control group A (Tables 1, 2). These results indicated that the model rats were in the state of serious heat syndrome and mild blood stasis syndrome (that is EHLBS). Compared with control group B, significantly elevated whole blood viscosities at all shear stresses (*p* < 0.01), prolonged APTT (*p* < 0.01) and TT (*p* < 0.01), and shortened PT (*p* < 0.01) were observed in the BS group (Table 3). These results demonstrated that the blood stasis aggravated in model rats. Histopathological features of the liver and lung tissues from the BS group were clearly different from those of control group B. In the BS group, structural disorder of hepatic lobules and erythrocytes clustered in the hepatic sinus and central vein of the hepatic lobules were observed (Figure 2). Several erythrocytes emerged in the small pulmonary veins and alveolar wall capillaries, and the alveolar space was filled with a few red blood cells (Figure 3). These findings confirmed that the model rats were in the BS stage.

**TABLE 1:** Comparison of inflammatory indices among different groups of the rats in EHLBS stage ( ‾x ± s, *n* = 10).

Group	IL-6	TNF-α	Forehead temperature	
			0 h (°C)	2 h (°C)
**Control group A**	107.02 ± 24.41	197.16 ± 15.90	36.49 ± 0.16	36.45 ± 0.14
**EHLBS group**	3,108.70 ± 345.99^**^	263.58 ± 26.31^**^	36.45 ± 0.17	37.53 ± 0.34^**^
**PRR-treated group A**	2019.07 ± 287.78^△△^	240.67 ± 19.00^△^	36.37 ± 0.13	37.02 ± 0.21^△△^

EHLBS v.s. Control group A ^**^p < 0.01; PRR-treated group A v.s. EHLBS ^△△^p < 0.01, ^△^p < 0.05.

**TABLE 2:** Comparison of hemorheological indices and coagulation indices among different groups of the rats in EHLBS stage ( ‾x ± s, *n* = 10).

Group	Hemorheological indices			Coagulation indices		
	1s^−1^(mPa’s)	50 s^−1^(mPa’s)	200 s^−1^(mPa’s)	PT	TT	APTT
**Control group A**	21.04 ± 0.44	4.35 ± 0.32	3.11 ± 0.24	13.04 ± 0.75	38.88 ± 6.52	27.15 ± 2.05
**EHLBS group**	21.75 ± 0.40^*^	4.81 ± 0.41^*^	3.69 ± 0.45^*^	13.07 ± 0.42	49.30 ± 2.32	39.47 ± 7.11^**^
**PRR-treated group A**	21.32 ± 0.45^△^	4.75 ± 0.35	3.45 ± 0.40	13.91 ± 1.55	54.18 ± 2.51	52.45 ± 11.32^△△^

EHLBS v.s. Control group A ^*^p < 0.05, ^**^p < 0.01; PRR-treated group A v.s. EHLBS ^△^p < 0.05, ^△△^p < 0.01.

**TABLE 3:** Comparison of hemorheological indices and coagulation indices among different groups of the rats in BS stage ( ‾x ± s, *n* = 10).

Group	3s^−1^ (mPa’s)	50 s^−1^ (mPa’s)	200 s^−1^ (mPa’s)	PT	APTT	TT
**Control group B**	10.06 ± 1.37	2.97 ± 0.27	2.52 ± 0.25	13.47 ± 0.13	21.18 ± 2.92	38.86 ± 2.83
**BS group**	19.80 ± 3.98^**^	4.37 ± 0.64^**^	3.62 ± 0.59^**^	12.49 ± 0.17^**^	27.64 ± 1.54^**^	46.64 ± 4.98^**^
**PRR-treated group B**	12.79 ± 1.38^△△^	3.65 ± 0.24^△△^	3.12 ± 0.42^△^	12.93 ± 0.35^△^	27.52 ± 3.30	46.25 ± 5.27

BS v.s. Control group B^**^p < 0.01; PRR-treated group B v.s. BS ^△△^p < 0.01, ^△^p < 0.05.

Next, the pharmacodynamic effects of PRR in treating EHLBS and BS were examined. Compared with the EHLBS group, the levels of inflammatory cytokines (TNF-α and IL-6), forehead temperature, and whole blood viscosity at 1 s^−1^ mPa’s (*p < 0.01, p < 0.05, p < 0.05,* and *p < 0.05*, respectively) were significantly attenuated to normal values, whereas APTT was further prolonged in PRR-treated group A (Tables 1, 2). PRR alleviates inflammation and improves hemorheology to treat EHLBS. Because inflammation was the most significant symptom, the anti-inflammatory effect was the most important pharmacodynamic effect of PRR in treating EHLBS. In addition, PRR had potent anticoagulant activity that could prolong APTT in both model and normal animals (Deng et al., 1991). Thus, we believe that APTT prolongation is a manifestation of the anticoagulant effect rather than a pharmacodynamic effect of PRR in treating EHLBS.

Compared with the BS group, the whole blood viscosities at 3, 50, and 200 s^−1^ mPa’s decreased to normal values and PT significantly prolonged to normal values (*p < 0.01, p < 0.01, p < 0.05,* and *p < 0.05*, respectively) in PRR-treated group B (Table 3). Moreover, the pathological injuries of the lung and liver tissues were markedly attenuated in PRR-treated group B. A relatively clearer alveolar wall structure and fewer erythrocytes in the small pulmonary veins, alveolar wall capillaries, and hepatic sinuses were observed in PRR-treated group B than in the BS group (Figures 2, 3). These findings indicate that PRR effectively treated BS by improving hemorheology and coagulation and alleviating hepatic and pulmonary injury.

According to TCM basic theory, PRR treats THBSS through clearing heat and promoting blood circulation. Modern pharmacological studies have found that the pharmacological basis of clearing heat includes anti-virus, anti-bacterial, regulating immunity, anti-inflammation, and relieve fever, etc., and the improvement of hemodynamic parameters, hemorheology, microcirculation, and anti-coagulation, etc. are the pharmacological basis of promoting blood circulation (Song 2009). Therefore, in this study, PRR exerted the efficacy of clearing heat and cooling blood through the improvement of inflammation, fever, and hemorheology to alleviate EHLBS; and to treat BS, PRR promoted blood circulation and removed blood stasis by improving hemorheology and coagulation. To the best of our knowledge, it was the first study to find that PRR could suppress the corresponding symptoms in both stages.

### 3.2 Elucidation of the Pharmacodynamic Mechanisms of PRR in Treating EHLBS and BS

#### 3.2.1 Regulatory Effects of Paeoniae Radix Rubra on Metabolic Profiles

**FIGURE 4:**
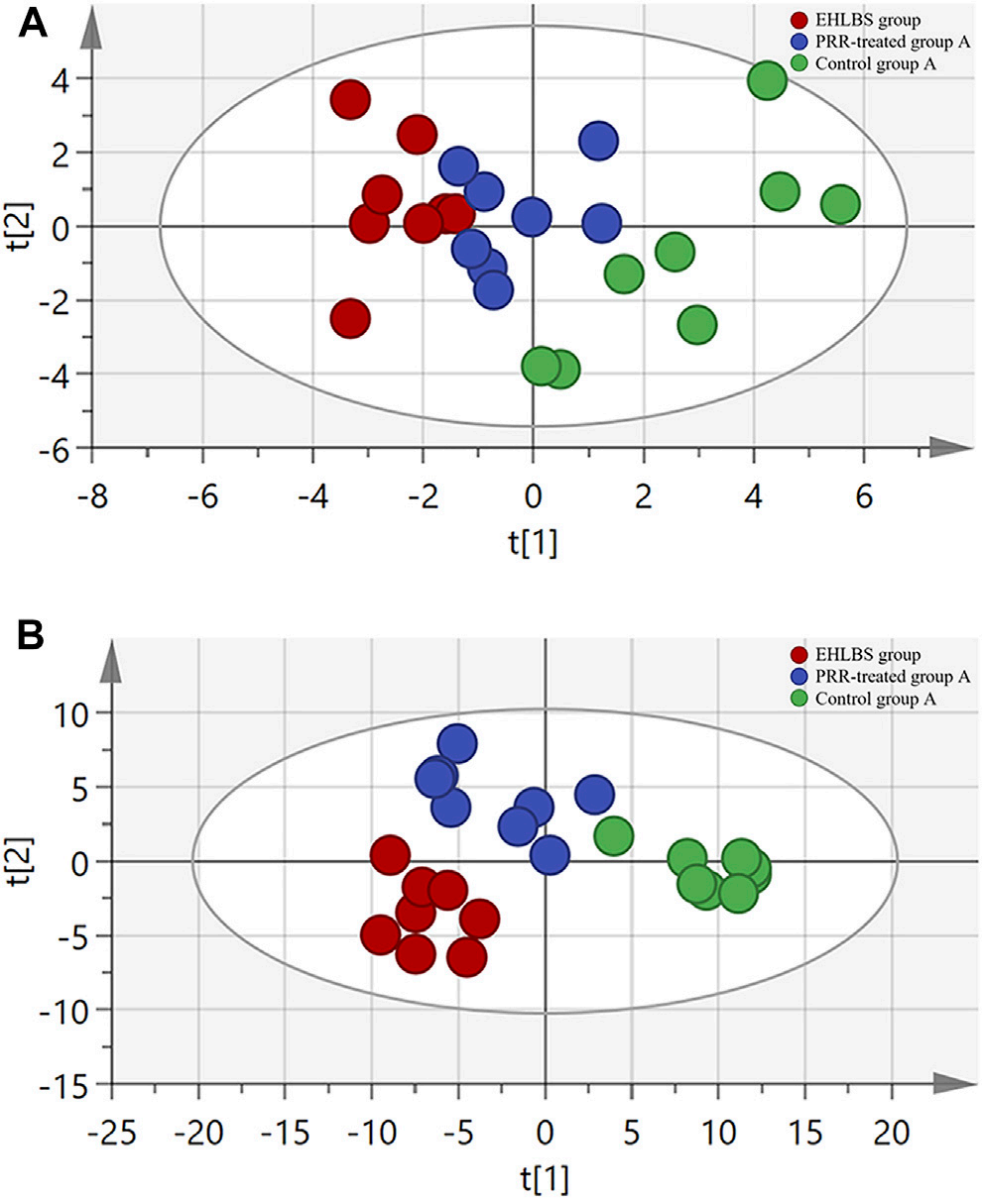
The relationship of metabolic phenotypes among three groups in EHLBS stage. **(A)** PCA plot. **(B)** PLS-DA plot. R^2^X and Q^2^Y of PCA are 0.693 and 0.751; R^2^X and Q^2^Y of PLS-DA are 0.705 and 0.869. The CV-ANOVA *p* value of PLS-DA was less than 0.05.

**FIGURE 5:**
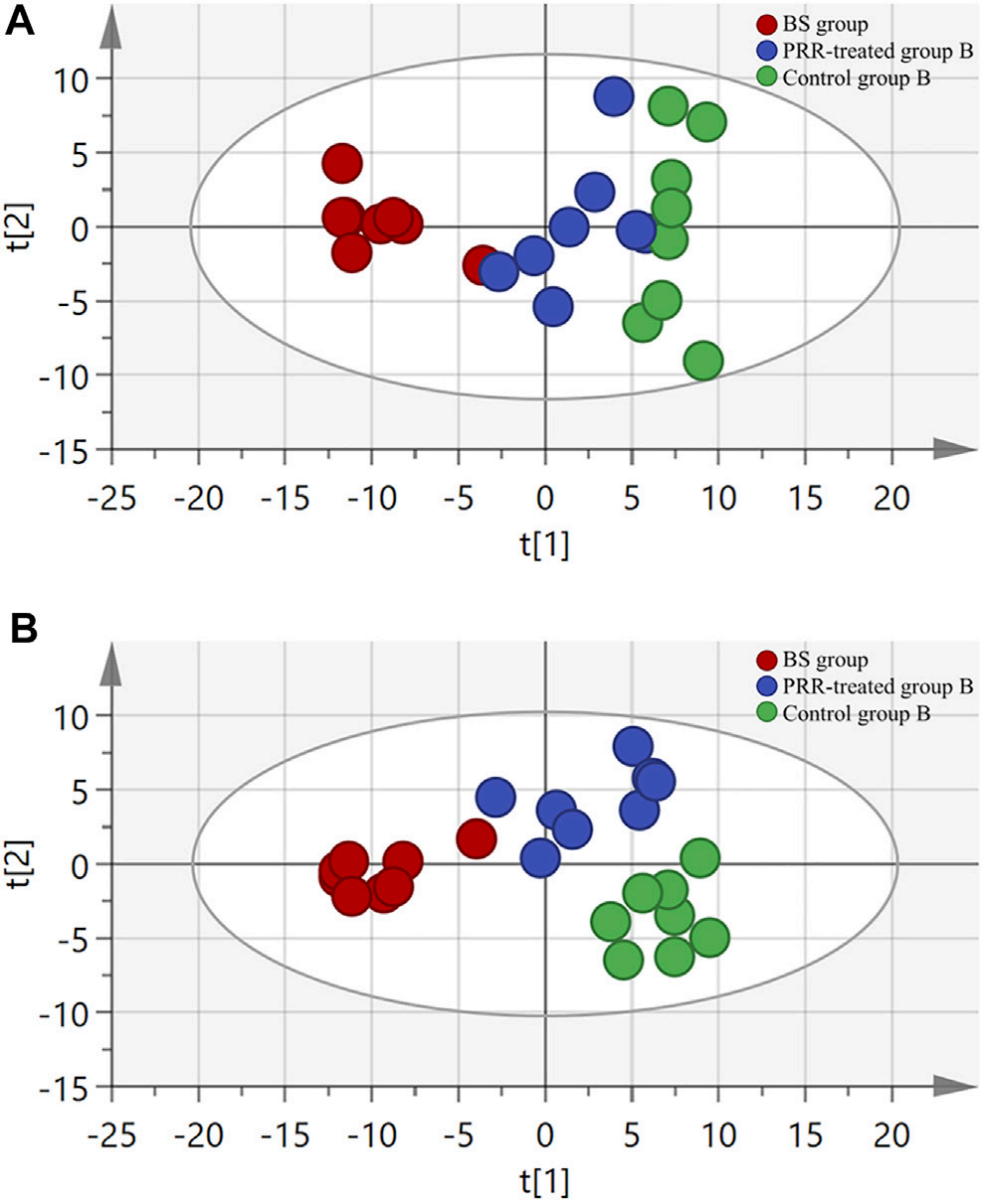
The relationship of metabolic phenotypes among three groups in BS stage. **(A)** PCA plot. **(B)** PLS-DA plot. R^2^X and Q^2^Y of PCA are 0.761 and 0.665; R^2^X and Q^2^Y of PLS-DA are 0.775 and 0.803. The CV-ANOVA *p* value of PLS-DA was less than 0.05.

Considering the important roles of amino acids, bile acids, and phospholipids in the progression of THBSS, to clarify the mechanisms of PRR in treating EHLBS and BS, we first analyzed these endogenous metabolites in rat plasma by targeted metabonomics. In total, 24 amino acids, 19 bile acids, and 133 phospholipids were assayed. Then, unsupervised PCA and supervised PLS-DA were conducted to explore whether PRR could improve the metabolic disorders in the EHLBS or BS stage. Figures 4, 5 show the score plots. In these score plots, the metabolites and their concentrations determine the relative positions of the samples. Therefore, the distribution of the samples reflects the similarity of the metabolic phenotypes among the groups. In both the EHLBS and BS stages, the control, model, and PRR-treated groups were separated, and the PRR-treated group was located much closer to the control group than the model group. The above results indicate that PRR could remarkably ameliorate the metabolic disturbances in these two stages.

#### 3.2.2 Remarkable Changes in Endogenous Metabolites and Related Metabolic Pathways Induced by Paeoniae Radix Rubra Treatment

In this study, we found that EHLBS induced significant changes in 105 endogenous metabolites, and BS induced significant changes in 69 endogenous metabolites (Supplementary Tables S4, S5). It was evident that the three categories of endogenous metabolites (i.e., amino acids, bile acids, and phospholipids) were significantly affected in both the EHLBS and BS stages. Among them, PRR attenuated the alterations of eight amino acids, one bile acid, seven phosphatidylcholines, two ceramides, and two lysophosphatides in the EHLBS stage (Table 4). Meanwhile, the levels of three bile acids, three lysophosphatides, four phosphatidylcholines, and two sphingomyelins were ameliorated by PRR in the BS stage (Table 5).

**TABLE 4:** 20 differential endogenous metabolites of the rats in EHLBS stage changed to normal level after PRR administration.

No	Metabolites	Category	Model/Control	PRR/Model
1	(3′-sulfo)galbeta-Cer(d18:1/16:0(2OH))	Phospholipid (ceramide)	↑*	↓*
2	cer(d18:0/14:0)	Ceramide	↑*	↓*
3	Alanine	Amino acid	↓*	↑*
4	Arginine	Amino acid	↓*	↑*
5	Citrulline	Amino acid	↓*	↑*
6	Glycine	Amino acid	↓*	↑
7	Histidine	Amino acid	↓*	↑*
8	Lysine	Amino acid	↓*	↑*
9	Ornithine	Amino acid	↓*	↑*
10	Serine	Amino acid	↓*	↑
11	PC(16:0/18:1(9Z))	Phospholipid (phosphatidylcholine)	↓*	↑*
12	PC(18:0/18:2(9Z,12Z))	Phospholipid (phosphatidylcholine)	↓*	↑
13	PC(18:2(9Z,12Z)/15:0)	Phospholipid (phosphatidylcholine)	↓*	↑
14	PC(18:2(9Z,12Z)/16:0)	Phospholipid (phosphatidylcholine)	↓*	↑*
15	PC(18:2(9Z,12Z)/17:0)	Phospholipid (phosphatidylcholine)	↓*	↑*
16	PC(20:4(5Z,8Z,11Z,14Z)/16:1(9Z))	Phospholipid (phosphatidylcholine)	↓*	↑*
17	PC(14:0/18:2(9Z,12Z))	Phospholipid (phosphatidylcholine)	↓*	↑
18	PC(22:0/0:0)	Phospholipid (lysophosphatide)	↓*	↑*
19	PC(O-18:0/0:0)	Phospholipid (lysophosphatide)	↓*	↑*
20	Taurodeoxycholic acid	Bile acid	↓*	↑*

Model/Control represented the comparison between EHLBS group and control group A; PRR/Model represented the comparison between PRR-treated group A and EHLBS group. ↓ denoted down-regulation; ↑ denoted up-regulation; *p < 0.05.

**TABLE 5:** 12 differential endogenous metabolites of the rats in BS stage changed to normal level after PRR administration.

No	Metabolites	Category	Model/Control	PRR/Model
1	Glycochenodeoxycholic acid	Bile acid	↓*	↑*
2	Glycodeoxycholic acid	Bile acid	↓*	↑*
3	Glycocholic acid	Bile acid	↓*	↑*
4	PC(0:0/18:0)	Phospholipid (lysophosphatide)	↑*	↓*
5	PC(16:0/16:0)	Phospholipid (phosphatidylcholine)	↑*	↓*
6	PC(18:0/16:0)	Phospholipid (phosphatidylcholine)	↑*	↓*
7	PC(18:0/18:2(9Z,12Z))	Phospholipid (phosphatidylcholine)	↑*	↓*
8	PC(22:6(4Z,7Z,10Z,13Z,16Z,19Z)/18:0)	Phospholipid (phosphatidylcholine)	↑*	↓*
9	PE(0:0/18:0)	Phospholipid (lysophosphatide)	↑*	↓*
10	PE(18:0/0:0)	Phospholipid (lysophosphatide)	↑*	↓*
11	SM(d16:1/16:0)	Phospholipid (sphingomyelin)	↑*	↓*
12	SM(d18:2/24:0)	Phospholipid (sphingomyelin)	↑*	↓*

Model/Control represented the comparison between BS group and control group B; PRR/Model represented the comparison between PRR-treated group B and BS group. ↓ denoted down-regulation; ↑ denoted up-regulation; *p < 0.05.

**FIGURE 6:**
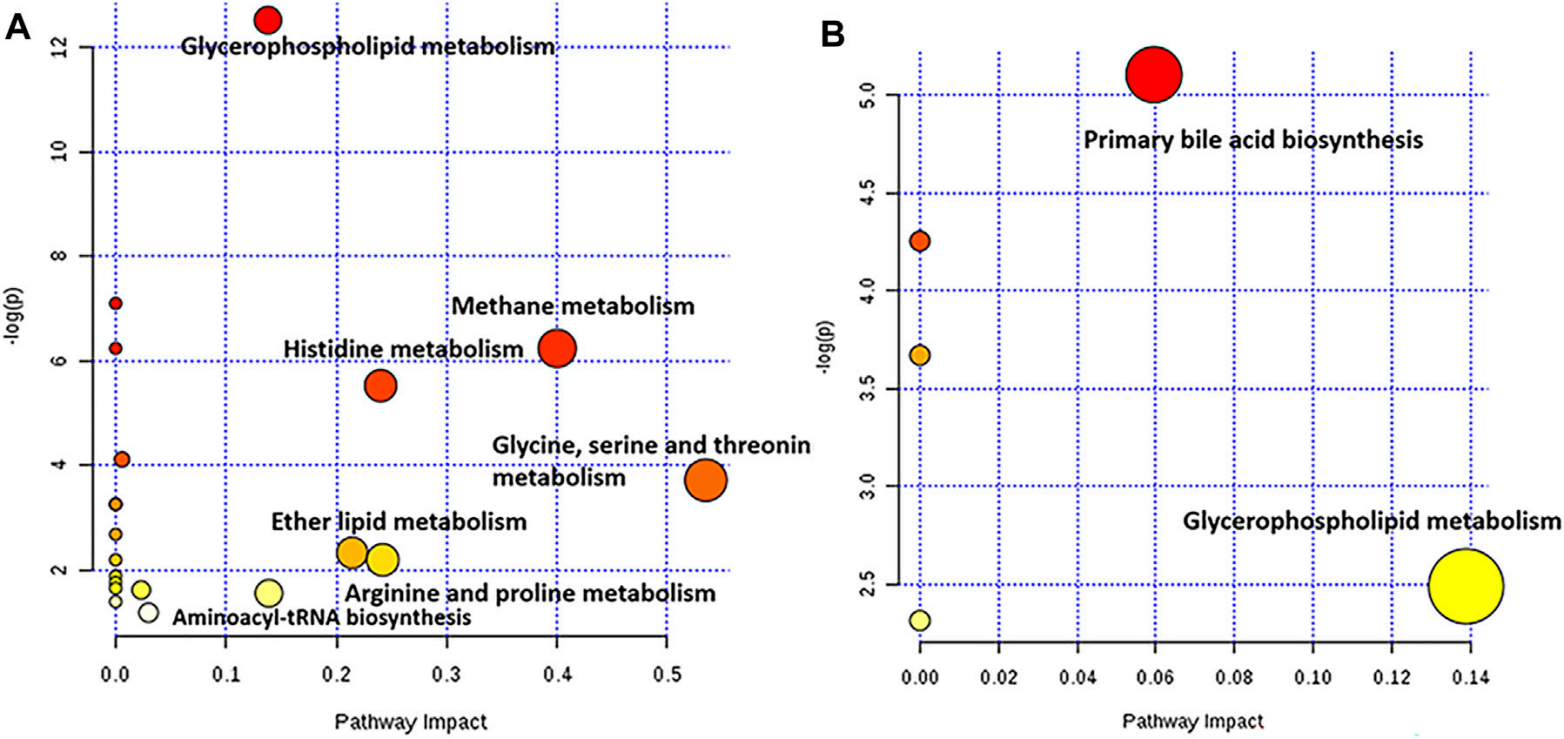
Pathway analysis with MetPA. **(A)** the summary of the pathways regulated by PRR in the EHLBS stage. **(B)** the summary of the pathways regulated by PRR in the BS stage. The size and color of each circle was based on pathway impact value and *p*-value, respectively. The darker of the circle demonstrates that the metabolic pathway is more significantly regulated by PRR. The larger of the circle represents that the more endogenous metabolites regulated by PRR in this metabolic pathway.

**FIGURE 7:**
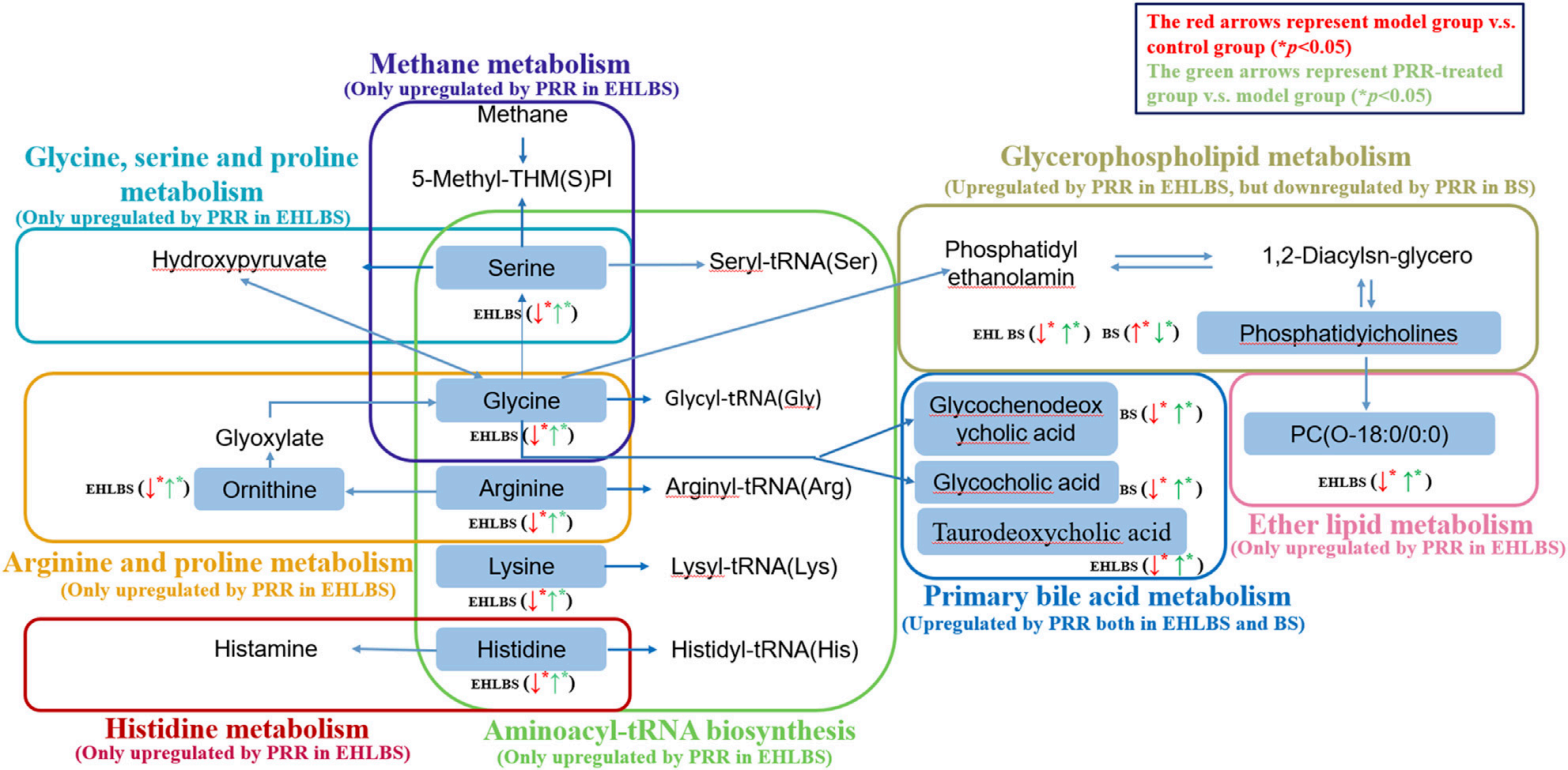
Metabolic networks associated with the pharmacodynamic effects of PRR in the EHLBS stage and BS stage. Eight dotted rectangles in different colors represented eight different pathways. Metabolites in the boxes were the endogenous metabolites PRR could regulate directly in this study. ↑denoted up-regulation; ↓denoted down-regulation.

Furthermore, a pathway analysis of the above-mentioned endogenous metabolites was conducted, and relevant metabolic pathways were identified (Supplementary Tables S8, S9). Summaries of the pathways and metabolic networks are shown in Figures 6, 7, respectively. In the EHLBS stage, PRR remarkably upregulated seven metabolic pathways, including glycerophospholipid metabolism, primary bile acid biosynthesis, and five amino acid metabolic pathways (such as arginine and proline metabolism). In the BS stage, PRR significantly downregulated glycerophospholipid metabolism and upregulated primary bile acid biosynthesis. However, PRR had no obvious effect on ether lipid metabolism and amino acid metabolism.

#### 3.2.3 Comparison of the Pharmacodynamic Mechanisms of Paeoniae Radix Rubra in Treating Excessive Heat and Little Blood Stasis and Blood Stasis Regulation of Amino Acid Metabolism

##### 3.2.3.1 Regulation of Amino Acid Metabolism

In the EHLBS stage, among the 20 endogenous metabolites whose alterations could be attenuated by PRR, eight were amino acids (Table 4). Specifically, PRR increased the levels of alanine, arginine, citrulline, glycine, histidine, lysine, ornithine, and serine, which were decreased in the EHLBS group. These eight amino acids primarily participated in five of the seven metabolic pathways (glycine, serine, and threonine metabolism; methane metabolism; histidine metabolism; arginine and proline metabolism; and aminoacyl-tRNA biosynthesis), with an impact value >0.1 (Supplementary Table S8). These results demonstrate that PRR significantly regulated amino acid metabolism and imply that PRR regulation of amino acid metabolism contributes greatly to the pharmacodynamic effects at this stage. However, in the BS stage, only four differential amino acids (glycine, valine, histidine, and taurine) were found between control group B and the BS group, and PRR could not attenuate their alterations (Supplementary Table S5). This implies that the pharmacodynamic effects of PRR in the BS stage have no obvious correlation with amino acid metabolism.

In the EHLBS stage, the contribution of the PRR-mediated upregulation of amino acids to the pharmacodynamic effects of PRR could be elucidated as follows.

Aminoacyl-tRNA biosynthesis is related to protein synthesis, and its pathway map involves alanine, arginine, glycine, histidine, lysine, and serine (Polycarpo et al., 2004). In our study, the variations in these amino acids suggest that the aminoacyl-tRNA biosynthesis metabolic pathway was downregulated in the EHLBS group but upregulated in PRR-treated group A. This indicates that PRR could enhance supplementation of amino acids for synthesizing acute phase proteins to clear harmful agents and aid tissue repair in the EHLBS stage.

Glycine, an inhibitory neurotransmitter in the central nervous system, is known to activate a glycine-gated chloride channel expressed in the membranes of inflammatory cells, including macrophages, monocytes, and T lymphocytes. Through inhibition of the activities of these cells, glycine can suppress the activation of transcription factors and prevent the formation of free radicals and inflammatory cytokines, thus eliciting anti-inflammatory activity. Additionally, glycine can generate serine, which can be converted into glucose for energy (Wheeler et al., 1999). We previously noted that energy is needed for the proliferation of inflammatory cells during this stage (Xu et al., 2017). Therefore, we considered that the elevation of glycine and serine in PRR-treated group A observed in the present study may be associated with preventing the expression of inflammatory cytokines.

In the arginine and proline metabolism pathway, arginine can generate ornithine and citrulline. Since arginine is the major substrate for nitric oxide synthesis, it plays an important role in immune responses. Arginine can attenuate liver damage and inflammation (Tian et al., 2013). Thus, the elevated levels of arginine, ornithine, and citrulline in PRR-treated group A observed in the present study suggest that the anti-inflammatory effect of PRR was related to improved arginine and proline metabolism.

Histidine is a common amino acid that is used by histidine decarboxylase to generate histamine, which in turn participates in inflammation and immune reactions. Histidine can suppress pro-inflammatory cytokine expression via the nuclear factor (NF)-κB pathway (Feng et al., 2013). Hence, the increase in histidine levels found in the present study in PRR-treated group A suggests that the regulation of histidine metabolism by PRR contributes to its anti-inflammatory effect.

Regarding the methane metabolism pathway, catalase can metabolize methanol into formaldehyde and then convert it to formic acid or formate, which has an important protective effect on peroxide toxicity. Zhao et al. (2018) reported that a decreased level of serine (a methane metabolism intermediate) was suggestive of reduced catalase activity, which might have led to an increased production of reactive oxygen species (ROS), and then through a series of signal transduction pathways, induced an inflammatory reaction. Therefore, the results of our study that PRR elevated the level of serine suggest that PRR can alleviate inflammation by preventing ROS formation.

Overall, the present study indicated that PRR can exert anti-inflammatory effects by upregulating amino acid metabolism in the EHLBS stage.

##### 3.2.3.2 Regulation of Bile Acid Metabolism

In both stages, PRR elevated the levels of bile acids that were reduced in the rat models. Specifically, PRR increased the level of taurodeoxycholic acid in the EHLBS stage, and the levels of glycochenodeoxycholic acid, glycodeoxycholic acid, and glycocholic acid in the BS stage (Tables 4, 5). Bile acids can activate the farnesyl X receptor to produce anticoagulant effects through the activation of protein C, inhibition of thrombin activity, and acceleration of thrombin inactivation (Wang et al., 1999). Therefore, the results of our study that PRR upregulated bile acids during these two stages indicate that PRR can activate the farnesyl X receptor to prolong coagulation time, which is consistent with the biochemical data (Tables 2, 3). Thus, we believe that the anticoagulant activity of PRR is attributable to its upregulation of bile acid metabolism.

##### 3.2.3.3 Regulation of Phospholipid Metabolism

We observed that PRR had complex effects on phospholipid metabolism when comparing the EHLBS and BS stages (Supplementary Tables S6, S7).

In the EHLBS stage, PRR upregulated glycerophospholipid metabolism and ether lipid metabolism resulting in increased levels of seven phosphatidylcholines and two lysophospholipids in the rat models (Supplementary Tables S8).

Phosphatidylcholines are intermediates of glycerophospholipid metabolism and indirectly reflect ether lipid metabolism. They are hydrolyzed to lysophosphatides, which play important roles in maintaining the integrity of membrane structure and function (Volinsky et al., 2016). Therefore, the elevated levels of phosphatidylcholines and lysophosphatides observed in our study suggest that PRR can repair cell membrane damage and reduce cell damage by upregulating glycerophospholipid metabolism and ether lipid metabolism.

In the EHLBS stage, we also found that PRR could reduce two ceramides, which were increased in the model group (Table 4). It is widely known that ceramide is the center of sphingolipid metabolism. Inflammatory cytokines can elevate ceramide levels, and ceramide can induce an inflammatory response through toll-like receptor-dependent and -independent mechanisms (Wei et al., 2019). Therefore, the anti-inflammatory activity of PRR in this study could be related to a decrease in ceramide levels.

In the BS stage, PRR downregulated glycerophospholipid metabolism resulting in decreased levels of four phosphatidylcholines, three lysophosphatides, and two sphingomyelins in the rat models (Supplementary Tables S9).

Phosphatidylcholines involved in glycerophospholipid metabolism are known to be hydrolyzed to lysophosphatides. The elevation in lysophosphatide levels results in a change in the red blood cells from normal disc-shaped erythrocytes into acanthocytes, which in turn reduces erythrocyte deformability and increases whole blood viscosity (Karliner 2002). Therefore, the reduction in whole blood viscosity by PRR in the BS stage in our study could be attributed to the downregulation of glycerophospholipid metabolism.

Sphingomyelins are signaling molecules that participate in many important cellular processes related to immune responses (Dai et al., 2016). Thus, the decrease in sphingomyelins observed in PRR-treated group B in our study suggests that PRR can alleviate immune damage during the BS stage.

In summary, PRR elevated bile acid levels in both the EHLBS and BS stages. However, it had different regulatory effects on amino acid and phospholipid metabolism in these two stages. Additionally, the PRR-mediated regulation of amino acid metabolism, bile acid metabolism, and phospholipid metabolism is closely related to its different pharmacodynamic effects in these two stages.

### 3.3 Effective Substances Study

The constituents of PRR extract and their metabolites in rat plasma were screened for potential effective substances related to the pharmacodynamic effects of PRR in the EHLBS and BS stages.

#### 3.3.1 Identification of the Original Constituents of Paeoniae Radix Rubra and Their Metabolites in Rat Plasma

The original constituents of PRR and their metabolites in rat plasma were identified using previously described methods (Liang et al., 2013). Regarding original constituents, eight compounds were found in each rat in both the EHLBS and BS stages, namely glucopyranosyl-paeonisuffrone (A1); mudanpioside F (A2); desbenzoylpaeoniflorin (A3); desbenzoylpaeoniflorin isomer II (A4); paeoniflorin (A5); oxypaeoniflorin (A6); 3,7- or 3,8-dimethyl ellagic acid (A7); and 4-*O*-methyldesbenzoylpaeoniflorin (A8) (Supplementary Table S10).

Based on the MS and MS^n^ data, 22 and 15 metabolites of PRR were tentatively identified in each rat in the EHLBS and BS stages, respectively (Supplementary Table S11). For example, the molecular formulae of M3 and M19 were determined to be C_10_H_12_O_7_S based on the ion at *m/z* 275.02. In their MS^2^ spectra, the [aglycone−H]^−^ at *m/z* 195.06 (C_10_H_11_O_4_) formed by the neutral loss of 79.96 Da (elemental composition: SO_3_) was observed, which had an additional CH_2_ unit in comparison with [aglycone−H]^−^ of 3,4-dihydroxy phenylpropionic acid (M2). This indicated that the aglycones of M3 and M19 were methylation products of 3,4-dihydroxy phenylpropionic acid. The C-3-OH is the preferred methylation site (Liang et al., 2013). Accordingly, M3, which had a larger peak area of 1,724,130, was identified as 3-methoxyl-4-hydroxy-phenylpropionic acid sulfate, and M19, which had a peak area of 1,105,485, was identified as 3-hydroxy-4-methoxyl-phenylpropionic acid sulfate (Supplementary Figure S2).

#### 3.3.2 Screening Potential Effective Substances of Paeoniae Radix Rubra in Treating Excessive Heat and Little Blood Stasis and Blood Stasis

PLSR was employed to discover effective substances in PRR in the treatment of EHLBS or BS by exploring the relationships between the differences in the peak areas of the original constituents and their metabolites and the resulting differences in pharmacodynamic effects (Gegentana et al., 2020). The peak areas of the original constituents and their metabolites were determined based on the extracted ion chromatograms (EICs). As mentioned in section 3.1, anti-inflammation was the main pharmacodynamic effect of PRR in treating EHLBS. Additionally, PRR mainly improved hemorheology and coagulation in the BS stage. Therefore, to screen for potential effective substances of PRR in treating EHLBS, the peak areas of the original constituents and their metabolites were selected as the independent variables (X), and both IL-6 and TNF-α levels were selected as the dependent variables (Y). To screen for potential effective substances in treating BS, X denoted the peak areas of the original constituents and their metabolites, and Y denoted the values of whole blood viscosity and PT. The PLS regression equations that were established had satisfactory goodness of fit and predictability (Supplementary Table S12).

According to the regression coefficients and variable importance in projection (VIP) values of the independent variables, potential effective substances of PRR in treating EHLBS or BS were found. An independent variable with a VIP value greater than 1 made a significant contribution to the dependent variable. A regression coefficient of less than 0 indicated a negative correlation between the independent and dependent variables. Conversely, a regression coefficient larger than 0 indicated a positive correlation between the independent and dependent variables.

For the EHLBS stage, 13 compounds (VIP>1, regression coefficient<0) related to the reduction in IL-6 levels were found: M15, M21, M20, M14, M11, M17, M12, M18, M6, M16, M3, A4, and M13 (Supplementary Table S13), and four compounds (VIP>1, regression coefficient<0) contributed to the reduction in TNF-α levels: A7, A5, A6, and M19 (Supplementary Table S14). Thus, 17 potential effective substances for treating EHLBS were found (Table 6). As alleviating inflammation is one of the manifestations of clearing heat, so these 17 compounds were also the potential effective substances of PRR to clear heat. Except for A4–A7, to the best of our knowledge, this is the first time that the other 13 compounds have been reported as potential effective substances of PRR.

**TABLE 6:** The information of 26 potential effective substances of PRR screened out in EHLBS stage and BS stage.

Syndrome stage	Compound	Formula	Identification result
**EHLBS stage**	A4	C_16_H_24_O_10_	Desbenzoylpaeoniflorin isomer II
	A5	C_23_H_28_O_11_	Paeoniflorin
	A6	C_23_H_28_O_12_	Oxypaeoniflorin
	A7	C_16_H_10_O_8_	3,7- or 3,8-dimethyl ellagic acid
	M3*	C_10_H_12_O_7_S	3-hydroxy-4-methoxy-phenylpropionic acid sulfate
	M6*	C_7_H_6_O_6_S	3- or 4-hydroxy benzoic acid sulfate
	M11*	C_16_H_26_O_8_	C_10_H_18_O_2_ glucuronide
	M12*	C_16_H_26_O_8_	C_10_H_18_O_2_ glucuronide
	M13*	C_16_H_26_O_8_	C_10_H_18_O_2_ glucuronide
	M14*	C_16_H_26_O_8_	C_10_H_18_O_2_ glucuronide
	M15*	C_16_H_26_O_8_	C_10_H_18_O_2_ glucuronide
	M16*	C_16_H_26_O_8_	C_10_H_18_O_2_ glucuronide
	M17*	C_16_H_26_O_10_	C_10_H_18_O_4_ glucuronide
	M18*	C_16_H_22_O_9_	C_10_H_14_O_3_ glucuronide
	M19*	C_10_H_12_O_7_S	3-methoxy-4-hydroxy-phenylpropionic acid sulfate
	M20*	C_14_H_16_O_9_	C_8_H_8_O_3_ glucuronide
	M21*	C_14_H_16_O_9_	C_8_H_8_O_3_ glucuronide
**BS stage**	M1	C_22_H_24_O_12_	3′-*O*-methyl (epi) catechin 5*-O*-glucuronide
	M2*	C_9_H_10_O_6_S	3-hydroxy phenylpropionic acid sulfate
	M11*	C_16_H_26_O_8_	C_10_H_18_O_2_ glucuronide
	M13*	C_16_H_26_O_8_	C_10_H_18_O_2_ glucuronide
	M15*	C_16_H_26_O_8_	C_10_H_18_O_2_ glucuronide
	M16*	C_16_H_26_O_8_	C_10_H_18_O_2_ glucuronide
	M18*	C_16_H_22_O_9_	C_10_H_14_O_3_ glucuronide
	M22*	C_13_H_14_O_8_	Benzoyl glucuronide
	M23*	C_16_H_26_O_8_	C_10_H_18_O_2_ glucuronide

13 Compounds marked with an asterisk was firstly regarded as potential effective substances of PRR.

For the BS stage, M22, M2, and M16 (VIP>1, regression coefficient<0) were related to the reduction in whole blood viscosity (Supplementary Table S15), and there were seven compounds related to the elevation in PT values (VIP>1, regression coefficient>0): M23, M18, M15, M1, M11, M13, and M16 (Supplementary Table S16). Thus, nine potential effective substances for treating BS were found (Table 6). Due to the improvement of hemorheology and coagulation reflects the efficacy of promoting blood circulation and removing blood stasis, so these nine compounds were also the potential effective substances of PRR to promote blood circulation and remove blood stasis. To the best of our knowledge, this is the first time that eight of them (except M1) have been reported as potential effective substances of PRR.

#### 3.3.3 Correlation Between Endogenous Metabolites and Potential Effective Substances of Paeoniae Radix Rubra in the Treatment of Excessive Heat and Little Blood Stasis or Blood Stasis

Since most of the above potential effective substances are metabolites, the lack of reference compounds limited the experimental validation of their pharmacodynamic effects in treating EHLBS or BS. Therefore, to evaluate the regulatory effects of potential effective substances on metabolic disorders, Pearson correlation analysis was used to illustrate the relationships between the potential effective substances and endogenous metabolites related to the therapeutic effects of PRR on EHLBS or BS. A *p* value less than 0.05 indicated a significant correlation.

**FIGURE 8:**
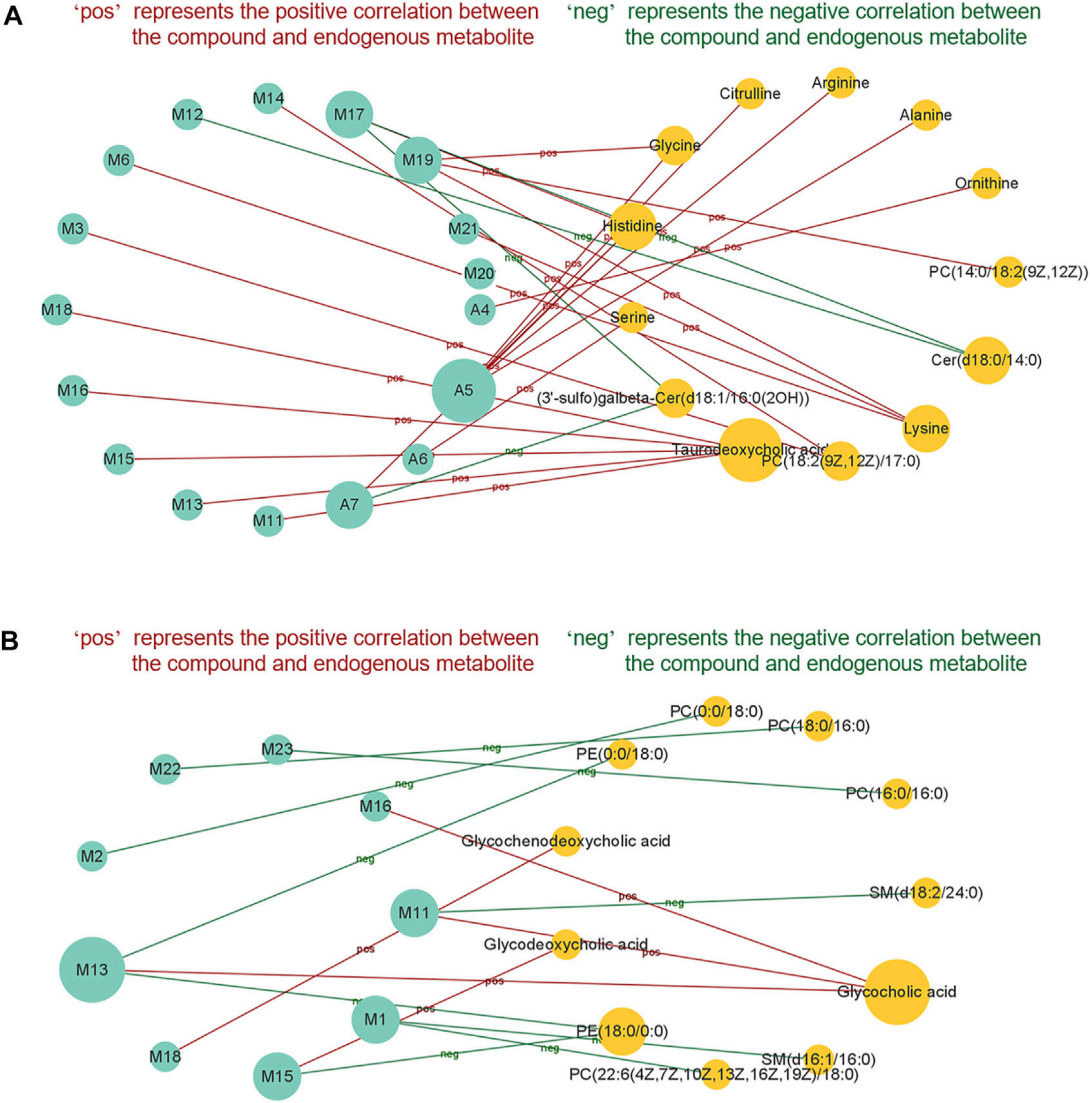
The correlationship between potential effective substances and endogenous metabolites in EHLBS stage **(A)** and BS stage **(B)**. Only potential effective substances and endogenous metabolites with significant correlation were shown in this figure. Green circle represents potential effective substance; orange circle represents endogenous metabolite. There is a significant correlation (*p* < 0.05) between the potential effective substance and endogenous metabolite, which are connected by a solid line.

In the EHLBS stage, the correlations between 20 endogenous metabolites whose abnormal changes could be attenuated by PRR (Table 4) and 17 potential effective substances (Table 6) were investigated. In the BS stage, the correlations between 12 endogenous metabolites whose abnormal changes could be attenuated by PRR (Table 5) and nine potential effective substances (Table 6) were studied. Figure 8 shows that 17 potential effective substances were significantly correlated with 13 endogenous metabolites in the EHLBS stage, and nine potential effective substances were significantly correlated with 11 endogenous metabolites in the BS stage. These findings indicate that the PRR-mediated regulations of the metabolic disorders caused by EHLBS or BS are inseparable from the corresponding potential effective substances.

From Figure 8, we also found that in the EHLBS stage, (3′-sulfo) Galbeta-Cer(d18:1/16:0(2OH)) and Cer(d18:0/14:0) were negatively correlated with A7, M12, and M17, while the other endogenous metabolites were positively correlated with the potential effective substances screened at this stage. As mentioned in the targeted metabonomics study (section 3.2.2), PRR downregulated (3′-sulfo) Galbeta-Cer(d18:1/16:0(2OH)) and Cer(d18:0/14:0), but upregulated the other endogenous metabolites in the EHLBS stage (Table 4).

Similarly, glycochenodeoxycholic acid, glycodeoxycholic acid, and glycocholic acid were positively correlated with M11, M13, M15, M16, and M18, while the other endogenous metabolites were negatively correlated with the potential effective substances screened in the BS stage (Figure 8). As found in the targeted metabonomics study (section 3.2.2), PRR upregulated glycochenodeoxycholic acid, glycodeoxycholic acid, and glycocholic acid, but downregulated the other endogenous metabolites in the BS stage (Table 5).

These results demonstrate that the relationship between the potential effective substances and endogenous metabolites was consistent with the trends in the regulation of these endogenous metabolites by PRR. As all of the potential effective substances that we found in section 3.3.2 were significantly correlated with the endogenous metabolites, we believe that the improvement in metabolic disorders observed in the EHLBS or BS stage with PRR treatment depends on these compounds. In other words, 17 and nine compounds that we found during PLSR screening of the EHLBS and BS stages, respectively, are more likely to be the material basis of PRR’s pharmacodynamic effects.

#### 3.3.4 Comparison of Potential Effective Substances of Paeoniae Radix Rubra in Treating Excessive Heat and Little Blood Stasis and Blood Stasis

Based on the comparison of potential effective substances screened in the EHLBS and BS stages, we found only five common compounds (Table 7). Among these five compounds, four (the isomers M11, M13, M15, and M16) were glucuronidation metabolites, whose aglycone structures are similar to that of a known drug with choleretic activity, epomediol (Supplementary Figure S3). Additionally, this drug is converted into a glucuronide to exhibit effects *in vivo* (Ventura et al., 1985), which indicates that these four isomers may also influence the levels of bile acids. Consistently, we found that M11, M13, M15, and M16 were significantly correlated with bile acid levels in the EHLBS and BS stages (Figure 8).

**TABLE 7:** The comparative analysis of potential effective substances of PRR in EHLBS stage and BS stage.

Types	Potential effective substances	
	EHLBS stage	BS stage
**The common compounds**	M11, M13, M15, M16, M18	
**The different compounds**	A4, A5, A6, A7, M3, M6, M12, M14, M17, M19, M20, M21	M1, M2, M22, M23

In addition, there were 12 potential effective substances specific to the treatment of EHLBS and four specific to the treatment of BS (Table 7). According to previous reports, desbenzoylpaeoniflorin isomer II (Yamahara 1982), paeoniflorin (Zhou et al., 2011), oxypaeoniflorin (Tani 1981), and 3,7- or 3,8-dimethyl ellagic acid (Cheng et al., 2011) (four specific compounds in treating EHLBS) possess anti-inflammatory activities. Additionally, as one of the specific compounds in treating BS, M1 (Claude et al., 2014) was previously shown to reverse endothelial dysfunction. These findings are in accordance with our pharmacodynamic evaluation results (Section 3.1), that is, the main pharmacodynamic effect of PRR in the EHLBS stage was an anti-inflammatory effect, whereas the main pharmacodynamic effect of PRR in the BS stage was to improve hemorheology and coagulation. Therefore, these results also reflect the reliability of the potential pharmacodynamic substances that we found in the two stages. The differences in the effective substances for the treatment of EHLBS and BS could be related to the different pharmacodynamic effects of PRR in these two stages.

## 4 Conclusion

Through using the stage-oriented strategy, the pharmacodynamic mechanisms and effective substances of PRR in the treatment of EHLBS and BS were elucidated for the first time. PRR treated EHLBS by upregulating primary bile acid biosynthesis, glycerophospholipid metabolism, ether lipid metabolism, and five amino acid metabolic pathways. Meanwhile, PRR could upregulate primary bile acid biosynthesis and downregulate glycerophospholipid metabolism to alleviate BS. 17 and nine potential effective substances were found in the EHLBS and BS stages, respectively. Among them, sixteen compounds were reported as potential effective substances of PRR for the first time. And there were only five common compounds between these two stages. These results demonstrated that the pharmacodynamic mechanisms and effective substances of PRR in the treatment of EHLB and BS were partly different. Therefore, this stage-oriented strategy is conducive to the elaboration of the pharmacodynamic mechanisms of TCD and the discovery of its effective substances. And it paves a new way to conduct future, related investigations. In clinical practice, TCM often treat diseases with prescriptions that combine several TCDs. Thus, in the future, we will study some such prescriptions containing PRR to further elucidate the pharmacodynamic mechanisms and effective substances of PRR.

## Data Availability

The original contributions presented in the study are included in the article/Supplementary Material, further inquiries can be directed to the corresponding authors.

## Abbreviations

APTT, activated partial thromboplastin time; BS, blood stasis; CAR, carrageenan; EHLBS, excessive heat and little blood stasis; EICs, extracted ion chromatograms; EVBCT, EDTA vacuum blood collection tubes; H-E, hematoxylin and eosin; LPS, lipopolysaccharide; PLSR, partial least squares regression; PRR, Paeoniae Radix Rubra; PT, prothrombin time; SCVBCT, sodium citrate vacuum blood collection tube; TCD, traditional Chinese drug; TCM, traditional Chinese medicine; THBSS, toxic heat and blood stasis syndrome; TT, thrombin time; VIP, variable important for projection.
