# Supplementary material for: Elucidation of the Mechanisms and Effective Substances of Paeoniae Radix Rubra Against Toxic Heat and Blood Stasis Syndrome With a Stage-Oriented Strategy
Source: Front Pharmacol. 2022 Mar 4;13:842839. doi: 10.3389/fphar.2022.842839 (PMC8931751; doi:10.3389/fphar.2022.842839)
Supplement: Supplementary file 1 [file DataSheet1.docx]

Supplementary Information 1

Chemical fingerprint of the PRR extract

To elucidate the chemical features of the PRR extract we used in this study, an HPLC method was established to determine its chemoprofile.

# Preparation of samples

32.8 mg PRR freeze-dried powder was dissolved in 10 mL water with ultrasound, then centrifuged at 8000 rpm for 10 min and the supernatant was filtered and injected for analysis.

# HPLC-based chemoprofile of the PRR extract

The separation was performed on an Agilent 1200 series (Agilent Corp., USA) equipped with diode array detector (DAD), using a Gemini NX C18 column (250 × 4.6 mm, i.d., 5 μm, Phenomenex Corp., USA). 20 μL of sample solution was injected into system and the column temperature was maintained at 25℃. The wavelength was set at 275 nm. The mobile phase was composed of 5% methanol - 0.1% formic acid aqueous solution (A) and acetonitrile (B) at a flow rate of 0.8 mL/min using a linear gradient elution program shown in Table S1. The HPLC-based chemoprofile of the PRR extract was shown in Figure S1.

**Table S1 HPLC gradient program of the PRR extract analysis**

| **Time (min)** | **Mobile phase B%** |
| --- | --- |
| 0-10 | 3-8 |
| 10-25 | 8-25 |
| 25-40 | 25-45 |
| 50-60 | 45-100 |


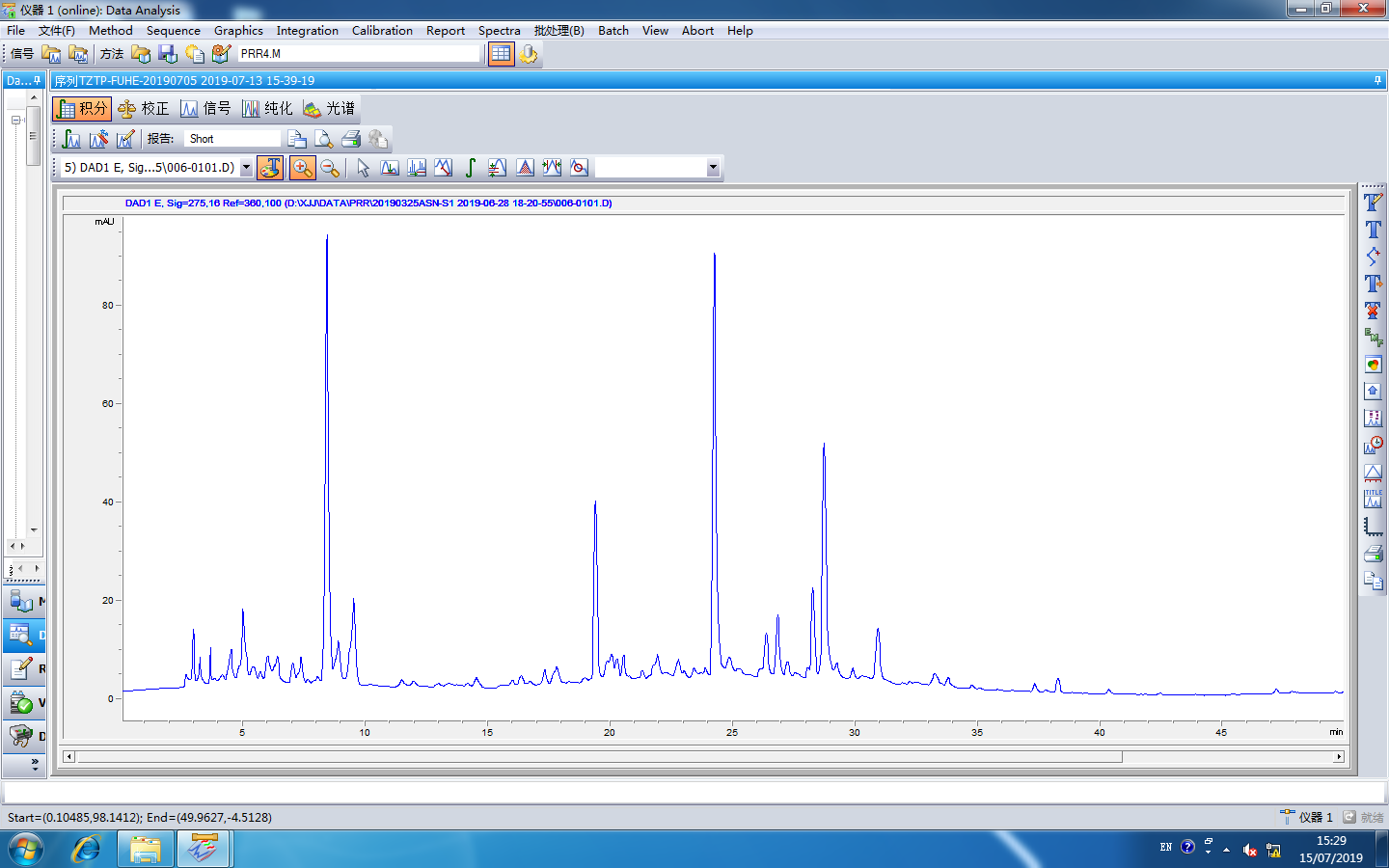


**Figure S1 Chemical fingerprint of the PRR extract using HPLC**

Supplementary Information 2

Determination of 5 major constituents in the PRR extract

To further ensure the reproducibility of the animal experiment results in this study, a UFLC-MS/MS method of simultaneous analysis of 5 major constituents (paeoniflorin, catechin, oxypaeoniflorin, benzoylpaeoniflorin, gallic acid) in the PRR was established and validated. Meanwhile, their contents in the PRR extract used in this study were measured.

# Materials and methods

# 1.1 Reagents and chemicals

Reference substances of paeoniflorin (PS000825), oxypaeoniflorin (PS010199), benzoylpaeoniflorin (PS000158) were purchased from Chengdu Push Bio-technology Co., Ltd (Chengdu, China). Catechin (A0158) and gallic acid (A0110) were purchased from Chengdu Must Biotechnology Co. Ltd (Chengdu, China).

LC-MS grade acetonitrile and HPLC grade methanol were purchased from Fisher Scientific (Fair Lawn, NJ, USA). Ultra-high purity water (18.2 MΩ, TOC< 5 ppb) was prepared by a Millipore Milli-Q Integral 3 Ultrapure Water System (Billerica, MA, USA). HPLC-grade formic acid was purchased from Tedia Company Inc. (Fairfield, OH, USA). Other chemicals were all analytical reagents.

PRR was purchased from the Beijing Tianheng pharmacy (Beijing, China; Lot no. 14701), and it was authenticated as the dried roots of *Paeonia lactiflora* Pall. by Dr. Feng Xu (School of Pharmaceutical Sciences, Peking University). A voucher sample (No. 7838) was deposited in the Herbarium of Pharmacognosy, School of Pharmaceutical Sciences, Peking University.

# 1.2 Preparation of standard solutions

Stock standard solutions of the 5 constituents were prepared with methanol and then diluted to different concentrations. All the stock standard solutions were stored at 4 °C in the refrigerator.

**1.3 LC-MS instruments and operation conditions**

**1.3.1 Liquid chromatography**

The separation was performed on an SHIMAZDU LC-30A System (Shimazdu Corp., Japan) using an Acquity UPLC HSS T3 column (100×2.1 mm, i.d., 1.8 μm, Waters, USA). A 2 μL sample was injected into system and the column temperature was maintained at 35°C. The mobile phase was composed of 0.1% formic acid aqueous solution (A) and acetonitrile (B) at a flow rate of 0.35 mL/min using a gradient elution of 15% B at 0-0.5 min, 15%-50% B at 0.5-3.5 min, 50%-90% B at 3.5-6.5 min, 90% B at 6.5-7 min, 90%-15% B at 7-7.5 min.

**1.3.2 Mass spectrometric conditions**

An 8050 triple quadrupole mass spectrometer (Shimadzu Corp., Japan) equipped with an electrospray ionization (ESI) interface was used for analytical detection. ESI/MS was operated in negative mode. The operating MS parameters were as follows: drying gas (N2) ﬂow rate, 10.0 L/min; nebulizing gas ﬂow rate, 3.0 l/min; heating gas ﬂow rate, 10.0 L/min; interface voltage, 3 kV; detector voltage, 1.8 kV; interface temperature, 300 °C; desolvation temperature, 250 °C; heat block temperature, 400 °C.

# Preparative method of PRR sample

32.8 mg PRR freeze-dried powder was dissolved in 10 mL water with ultrasound, then centrifuged at 8000 rpm for 10 min and the supernatant was filtered and injected for analysis.

# Method validation

**3.1 Linearity**

The linearity was assessed by assaying calibration curves with standard work solutions. Peak areas of analytes were recorded and calibration curves were drawn, then regression equations of the curves and correlation coefficients (r^2^) were calculated.

**3.2 Precision**

The precisions at three levels were evaluated by using the high-, medium- and low-level quality control samples prepared above.

**3.3 Stability**

Three concentrations of samples were assayed at 4, 12 and 24 h after the PRR extract was prepared. The peak areas of analytes were recorded. The averages and standard deviation (SD) values of concentrations for 5 constituents in 24 h were calculated and relative standard deviation (RSD%) was also calculated as an estimate of stability.

# Results

**4.1 Method validation**

The calibration curves, correlation coefficients, linear ranges, high-, medium-, low-concentration precision and stability within 24 h of the 5 analytes are showed in Tables S2 and S3. The calibration curves of 5 analytes exhibited good linearity within the selected ranges with the correlation coefficients (r2) between 0.997 and 0.999. All the results indicated that the established method had a satisfactory precision and stability.

**4.2 Contents of 5 constituents in the PRR extract**

The prepared sample of the PRR extract was injected and analyzed for 3 times. The areas of 5 analytes were recorded and contents were calculated with regression equations.

The contents of 5 analytes in the PRR extract were as follows: paeoniflorin 5.63 mg/g, catechin 0.13 mg/g, oxypaeoniflorin 0.41 mg/g, benzoylpaeoniflorin 0.13 mg/g, gallic acid 0.15 mg/g.

**Table S2 Molecular formula, quantification transition, retention time, calibration curve and correlation coefficient of 5 constituents in the PRR extract**

| **Constituent** | **Molecular formula** | **Quantification transition (m/z)** | **Retention time (t_R_, min)** | **Calibration curve** | **Correlation coefficient (r^2^)** |
| --- | --- | --- | --- | --- | --- |
| **Paeoniflorin** | C_23_H_27_O_11_ | 525.30>449.05 | 2.50 | y = 3.51×10^6^x + 1.57×10^6^ | 0.999 |
| [**Catechin**](http://www.baidu.com/link?url=XBgxXrWgdD3ul4mSwFRIApbHITHtHsqO-J70JFcsAWgD__pLWnx35TFQ869zvt7yw9_ePcI4_JsXOg8W4uDytxUH5ps_qlkHIocCBSBhcC3) | C_15_H_13_O_6_ | 289.20>245.10 | 1.85 | y = 1.91×10^6^x – 639.08 | 0.997 |
| **Oxypaeoniflorin** | C_23_H_27_O_12_ | 495.20>137.10 | 1.50 | y = 3.95×10^6^x + 189.21 | 0.999 |
| \|  \| [**Benzoylpaeoniflorin**](http://www.baidu.com/link?url=-Hg5ffzHsQF9FEMcivdufNNK-qIuaAnb9jWBPeJlDC_Sx2iV4jUlh2TS9ccZ5VCpEarUs0BVh-5zcCSVBqwkNcG1SJejpCaeYjNQJRxeHRhfhOE3Am5uCm6Zcq7nRvt5) \| \| --- \| --- \| | C_30_H_31_O_12_ | 629.35>121.05 | 3.98 | y = 3.76×10^6^x + 38.75 | 0.999 |
| [**Gallic acid**](http://www.baidu.com/link?url=eISCTQ2lfSLeFgAzdQFVy1X_qpFmlD9Nl5DRokvcQGHDoNZ2ClSWNSoGw78Vb28pGHd0aGBEYZxBO97ICn9bqvGWFcQJLlKk_JupL4CGSC_) | C_7_H_5_O_5_ | 169.20>125.05 | 1.02 | y = 635.32x + 55.48 | 0.999 |

**Table S3 Linear ranges, high-, medium- and low-concentration precisions and stability in 24 h of 5 constituents in the PRR extract**

| **Constituent** | **Linear ranges**  **(μg/ml)** | **Precisions** | | | | | | | **Stability in 24 h**  **(RSD)** |
| --- | --- | --- | --- | --- | --- | --- | --- | --- | --- |
|  |  | **High (ng/ml, %)** | | | **Medium (ng/ml, %)** | | **Low (ng/ml, %)** | |  |
| **Paeoniflorin** | 0.7812-25.00 | 12.50 | 99.78 | 3.125 | | 102.87 | 0.7812 | 99.20 | 1.14 |
| [**Catechin**](http://www.baidu.com/link?url=XBgxXrWgdD3ul4mSwFRIApbHITHtHsqO-J70JFcsAWgD__pLWnx35TFQ869zvt7yw9_ePcI4_JsXOg8W4uDytxUH5ps_qlkHIocCBSBhcC3) | 0.01560-0.5000 | 0.2500 | 104.40 | 0.06250 | | 100.27 | 0.01560 | 91.74 | 3.79 |
| **Oxypaeoniflorin** | 0.08825-2.825 | 1.413 | 103.50 | 0.3531 | | 103.55 | 0.8825 | 103.08 | 0.16 |
| **Benzoylpaeoniflorin** | 0.03280-1.050 | 0.5250 | 100.43 | 0.1312 | | 100.83 | 0.03280 | 94.51 | 0.74 |
| [**Gallic acid**](http://www.baidu.com/link?url=eISCTQ2lfSLeFgAzdQFVy1X_qpFmlD9Nl5DRokvcQGHDoNZ2ClSWNSoGw78Vb28pGHd0aGBEYZxBO97ICn9bqvGWFcQJLlKk_JupL4CGSC_) | 0.1625-5.200 | 2.600 | 100.81 | 0.6500 | | 100.02 | 0.1625 | 99.49 | 4.17 |

Supplementary Information 3

Targeted metabonomics analysis

# Analysis methods of targeted metabonomics

# Analysis of bile acids

The analysis was performed using a Dionex UltiMate 3000 LC system (Thermo Fisher Scientific, Massachusetts, USA) connected to a Q Exactive mass spectrometer (Thermo Fisher Scientific, Massachusetts, USA). Separation was performed on a Waters HSS T3 column (2.1 mm × 100 mm, 1.8 μm) kept at 40◦C and at a flow rate of 0.3 mL/min. The gradient mobile phase was a mixture of 0.1% formic acid in water (A) and acetonitrile (B). The gradient elution was performed as follows: 0–1.5 min, 0% B; 1.5–3.0 min, 0%–30% B; 3.0–6.0 min, 30%–50% B; 6.0–8.0 min, 50%–68% B; 8.0–15 min, 68%–88% B; 15–20 min, 88%–100% B. 1 μL of sample solution was injected for each run. During the whole analysis, all the samples were maintained at 15◦C. The spray voltage was 3700 V, evaporation temperature was 350°C, sheath gas was 28 arb., auxiliary gas was 8 arb., capillary temperature was 320°C, and S-lens RF was 50. The scan was in negative mode and the scan range was m/z 100–1500.

# Analysis of lipids

The analysis was performed using a Dionex UltiMate 3000 LC system (Thermo Fisher Scientific, Massachusetts, USA) connected to a Q Exactive mass spectrometer (Thermo Fisher Scientific, Massachusetts, USA). Separation was performed on a Waters CSH C18 column (1.7 mm × 100 mm, 1.8 μm) kept at 40◦C and at a flow rate of 0.3 mL/min. The gradient mobile phase was a mixture of 0.1% formic acid and 2 mmol/L ammonium formate in water (A) and methanol (B). The gradient elution was performed as follows: 0–6.0 min, 75% B; 6.0–15.0 min, 75%–100% B. 1 μL of sample solution was injected for each run. During the whole analysis, all the samples were maintained at 15◦C. The spray voltage was 3700 V, evaporation temperature was 350°C, sheath gas was 28 arb., auxiliary gas was 8 arb., capillary temperature was 320°C, and S-lens RF was 50. The scan was in negative mode and the scan range was m/z 100–1500.

# Analysis of amino acids

The analysis was performed using a Shimadzu LC 20 AD system (Shimadzu, Kyoto, Japan) connected to a 5500 Q-trap mass spectrometer (AB Sciex, Redwood, USA). Separation was performed on an Acquity BEH amide column (1.7 mm × 100 mm, 1.8 μm) kept at 50◦C and at a flow rate of 0.3 mL/min. The gradient mobile phase was a mixture of 0.1% formic acid and 2.5 mmol/L ammonium formate in water (A) and acetonitrile (B). The gradient elution was performed as follows: 0–6.0 min, 75% B; 6.0–15.0 min, 75%–100% B. 1 μL of sample solution was injected for each run. During the whole analysis, all the samples were maintained at 15◦C. The curtain gas was 40 kPa, collision gas was medium, ionspray voltage was 1500 V, nebulizer gas was 50 kPa, heater gas was 60 kPa, declustering potential was 10 V and ionization temperature was 600◦C. The scan was in negative mode and the scan range was m/z 100–1500.

# Results

The differential metabolites between the groups were shown in the Tables S4-S7. The relevant metabolic pathways were listed in Tables S8 and S9.

**Table S4 105 differential endogenous metabolites between the** **control group A and the EHLBS group**

| **No.** | **Endogenous metabolites** | **Category** | **Content variance** | **Trend** |
| --- | --- | --- | --- | --- |
| **1** | Tauro-*α*-muricholic acid | Bile acid | 1.98 | ↑* |
| **2** | Taurocholic acid | Bile acid | 2.84 | ↑* |
| **3** | Taurodeoxycholic acid | Bile acid | 0.077 | ↓* |
| **4** | Hyodeoxycholtaurine | Bile acid | 1.75 | ↑* |
| **5** | Lithocholyltaurine | Bile acid | 39155 | ↑* |
| **6** | Tauroursodeoxycholic acid | Bile acid | 2.26 | ↑* |
| **7** | Deoxycholic acid | Bile acid | 2.31 | ↑* |
| **8** | PC(0:0/14:0) | Phospholipid (lysophosphatide) | 0.56 | ↓* |
| **9** | PC(0:0/16:0) | Phospholipid (lysophosphatide) | 0.77 | ↓* |
| **10** | PC(0:0/16:1(9Z)) | Phospholipid (lysophosphatide) | 0.42 | ↓* |
| **11** | PC(0:0/17:0) | Phospholipid (lysophosphatide) | 0.82 | ↓* |
| **12** | PC(0:0/18:1(9Z)) | Phospholipid (lysophosphatide) | 0.62 | ↓* |
| **13** | PC(0:0/18:2(9Z,12Z)) | Phospholipid (lysophosphatide) | 0.47 | ↓* |
| **14** | PC(0:0/18:3(9Z,12Z,15Z)) | Phospholipid (lysophosphatide) | 0.83 | ↓* |
| **15** | PC(0:0/20:2(11Z,14Z)) | Phospholipid (lysophosphatide) | 0.63 | ↓* |
| **16** | PC(0:0/20:4(5Z,8Z,11Z,14Z)) | Phospholipid (lysophosphatide) | 0.63 | ↓* |
| **17** | PC(0:0/20:5(5Z,8Z,11Z,14Z,17Z)) | Phospholipid (lysophosphatide) | 0.46 | ↓* |
| **18** | PC(0:0/22:6(4Z,7Z,10Z,13Z,16Z,19Z)) | Phospholipid (lysophosphatide) | 0.58 | ↓* |
| **19** | PC(14:0/0:0) | Phospholipid (lysophosphatide) | 0.56 | ↓* |
| **20** | PC(16:0/0:0) | Phospholipid (lysophosphatide) | 0.86 | ↓* |
| **21** | PC(16:1(9Z)/0:0) | Phospholipid (lysophosphatide) | 0.38 | ↓* |
| **22** | PC(17:0/0:0) | Phospholipid (lysophosphatide) | 0.82 | ↓* |
| **23** | PC(18:1(9Z)/0:0) | Phospholipid (lysophosphatide) | 0.59 | ↓* |
| **24** | PC(18:2(9Z,12Z)/0:0) | Phospholipid (lysophosphatide) | 0.59 | ↓* |
| **25** | PC(20:0/0:0) | Phospholipid (lysophosphatide) | 0.64 | ↓* |
| **26** | PC(20:2(11Z,14Z)/0:0) | Phospholipid (lysophosphatide) | 0.61 | ↓* |
| **27** | PC(20:4(5Z,8Z,11Z,14Z)/0:0) | Phospholipid (lysophosphatide) | 0.78 | ↓* |
| **28** | PC(20:5(5Z,8Z,11Z,14Z,17Z)/0:0) | Phospholipid (lysophosphatide) | 0.61 | ↓* |
| **29** | PC(22:0/0:0) | Phospholipid (lysophosphatide) | 0.65 | ↓* |
| **30** | PC(22:4(7Z,10Z,13Z,16Z)/0:0) | Phospholipid (lysophosphatide) | 0.50 | ↓* |
| **31** | PC(22:6(4Z,7Z,10Z,13Z,16Z,19Z)/0:0) | Phospholipid (lysophosphatide) | 0.71 | ↓* |
| **32** | PC(18:3(9Z,12Z,15Z)/0:0) | Phospholipid (lysophosphatide) | 0.91 | ↓* |
| **33** | PE(0:0/16:0) | Phospholipid (lysophosphatide) | 0.70 | ↓* |
| **34** | PE(0:0/18:0) | Phospholipid (lysophosphatide) | 0.86 | ↓* |
| **35** | PE(16:0/0:0) | Phospholipid (lysophosphatide) | 0.75 | ↓* |
| **36** | PE(18:1(9Z)/0:0) | Phospholipid (lysophosphatide) | 0.63 | ↓* |
| **37** | PE(18:2(9Z,12Z)/0:0) | Phospholipid (lysophosphatide) | 0.52 | ↓* |
| **38** | PE(20:4(5Z,8Z,11Z,14Z)/0:0) | Phospholipid (lysophosphatide) | 0.85 | ↓* |
| **39** | PE(22:4(7Z,10Z,13Z,16Z)/0:0) | Phospholipid (lysophosphatide) | 0.42 | ↓* |
| **40** | PE(0:0/18:1(9Z)) | Phospholipid (lysophosphatide) | 0.61 | ↓* |
| **41** | PE(0:0/18:2(9Z,12Z)) | Phospholipid (lysophosphatide) | 0.48 | ↓* |
| **42** | PE(0:0/22:6(4Z,7Z,10Z,13Z,16Z,19Z)) | Phospholipid (lysophosphatide) | 0.72 | ↓* |
| **43** | PC(O-16:0/0:0) | Phospholipid (lysophosphatide) | 0.79 | ↓* |
| **44** | PC(O-18:0/0:0) | Phospholipid (lysophosphatide) | 0.79 | ↓* |
| **45** | PC(18:0/22:5(7Z,10Z,13Z,16Z,19Z)) | Phospholipid (phosphatidylcholine) | 0.86 | ↓* |
| **46** | PC(14:0/18:2(9Z,12Z)) | Phospholipid (phosphatidylcholine) | 0.69 | ↓* |
| **47** | PC(18:1(9Z)/17:0) | Phospholipid (phosphatidylcholine) | 0.78 | ↓* |
| **48** | PC(16:0/17:1(9Z)) | Phospholipid (phosphatidylcholine) | 0.71 | ↓* |
| **49** | PC(18:2(9Z,12Z)/15:0) | Phospholipid (phosphatidylcholine) | 0.70 | ↓* |
| **50** | PC(18:2(9Z,12Z)/16:0) | Phospholipid (phosphatidylcholine) | 0.87 | ↓* |
| **51** | PC(18:2(9Z,12Z)/17:0) | Phospholipid (phosphatidylcholine) | 0.87 | ↓* |
| **52** | PC(18:0/18:2(9Z,12Z)) | Phospholipid (phosphatidylcholine) | 0.82 | ↓* |
| **53** | PC(18:3(9Z,12Z,15Z)/16:0) | Phospholipid (phosphatidylcholine) | 0.46 | ↓* |
| **54** | PC(18:0/18:1(9Z)) | Phospholipid (phosphatidylcholine) | 0.83 | ↓* |
| **55** | PC(20:4(5Z,8Z,11Z,14Z)/14:0) | Phospholipid (phosphatidylcholine) | 0.77 | ↓* |
| **56** | PC(20:4(5Z,8Z,11Z,14Z)/15:0) | Phospholipid (phosphatidylcholine) | 0.83 | ↓* |
| **57** | PC(20:4(5Z,8Z,11Z,14Z)/16:1(9Z)) | Phospholipid (phosphatidylcholine) | 0.95 | ↓* |
| **58** | PC(20:4(5Z,8Z,11Z,14Z)/18:1(9Z)) | Phospholipid (phosphatidylcholine) | 0.92 | ↓* |
| **59** | PC(20:4(5Z,8Z,11Z,14Z)/18:2(9Z,12Z)) | Phospholipid (phosphatidylcholine) | 0.55 | ↓* |
| **60** | PC(16:0/18:1(9Z)) | Phospholipid (phosphatidylcholine) | 0.85 | ↓* |
| **61** | PC(20:0/18:1(9Z)) | Phospholipid (phosphatidylcholine) | 0.82 | ↓* |
| **62** | PC(16:1(9Z)/16:0) | Phospholipid (phosphatidylcholine) | 0.74 | ↓* |
| **63** | PC(20:3(8Z,11Z,14Z)/16:0) | Phospholipid (phosphatidylcholine) | 0.56 | ↓* |
| **64** | PC(22:6(4Z,7Z,10Z,13Z,16Z,19Z)/14:0) | Phospholipid (phosphatidylcholine) | 0.49 | ↓* |
| **65** | PC(22:6(4Z,7Z,10Z,13Z,16Z,19Z)/16:0) | Phospholipid (phosphatidylcholine) | 0.55 | ↓* |
| **66** | PE(18:0/18:1(9Z)) | Phospholipid (phosphatidylethanolamine) | 0.71 | ↓* |
| **67** | PC(O-16:0/16:0) | Phospholipid (plasmalogen) | 1.40 | ↑* |
| **68** | PC(O-16:0/18:3(9Z,12Z,15Z)) | Phospholipid (plasmalogen) | 1.52 | ↑* |
| **69** | PC(P-16:0/18:1(9Z)) | Phospholipid (plasmalogen) | 1.28 | ↑* |
| **70** | PC(P-18:0/18:2(9Z,12Z)) | Phospholipid (plasmalogen) | 0.76 | ↓* |
| **71** | PE(P-18:0/18:2(9Z,12Z)) | Phospholipid (plasmalogen) | 1.48 | ↑* |
| **72** | Etn-1-P-Cer(d14:1/18:0) | Phospholipid (ceramide) | 1.22 | ↑* |
| **73** | Cer(d18:0/14:0) | Phospholipid (ceramide) | 1.25 | ↑* |
| **74** | (3'-sulfo)Galbeta-Cer(d18:1/16:0(2OH)) | Phospholipid (ceramide) | 1.41 | ↑* |
| **75** | SM (d18:1/16:0) | Phospholipid (sphingomyelin) | 1.42 | ↑* |
| **76** | SM (d18:1/18:1(9Z)) | Phospholipid (sphingomyelin) | 1.39 | ↑* |
| **77** | SM(d16:0/20:0) | Phospholipid (sphingomyelin) | 15.03 | ↑* |
| **78** | SM(d16:1/16:0) | Phospholipid (sphingomyelin) | 1.32 | ↑* |
| **79** | SM(d16:1/17:0) | Phospholipid (sphingomyelin) | 1.22 | ↑* |
| **80** | SM(d16:1/18:0) | Phospholipid (sphingomyelin) | 1.42 | ↑* |
| **81** | SM(d16:1/20:1) | Phospholipid (sphingomyelin) | 1.39 | ↑* |
| **82** | SM(d16:1/24:1) | Phospholipid (sphingomyelin) | 1.24 | ↑* |
| **83** | SM(d18:0/16:0) | Phospholipid (sphingomyelin) | 2.50 | ↑* |
| **84** | SM(d18:1/18:0) | Phospholipid (sphingomyelin) | 1.56 | ↑* |
| **85** | SM(d18:1/20:0) | Phospholipid (sphingomyelin) | 1.83 | ↑* |
| **86** | SM(d18:2/18:1) | Phospholipid (sphingomyelin) | 2.61 | ↑* |
| **87** | SM(d18:2/20:0) | Phospholipid (sphingomyelin) | 1.45 | ↑* |
| **88** | SM(d18:2/24:0) | Phospholipid (sphingomyelin) | 1.14 | ↑* |
| **89** | SM(d18:2/24:1) | Phospholipid (sphingomyelin) | 1.17 | ↑* |
| **90** | SM(d19:1/20:0) | Phospholipid (sphingomyelin) | 1.58 | ↑* |
| **91** | Alanine | Amino acid | 0.58 | ↓* |
| **92** | Arginine | Amino acid | 0.63 | ↓* |
| **93** | Asparagine | Amino acid | 0.58 | ↓* |
| **94** | Aspartic acid | Amino acid | 0.69 | ↓* |
| **95** | Glutamine | Amino acid | 0.97 | ↓* |
| **96** | Glycine | Amino acid | 0.83 | ↓* |
| **97** | Histidine | Amino acid | 0.78 | ↓* |
| **98** | Lysine | Amino acid | 0.70 | ↓* |
| **99** | Methionine | Amino acid | 0.83 | ↓* |
| **100** | Proline | Amino acid | 0.48 | ↓* |
| **101** | Serine | Amino acid | 0.68 | ↓* |
| **102** | Tyrosine | Amino acid | 0.66 | ↓* |
| **103** | Ornithine | Amino acid | 0.61 | ↓* |
| **104** | Citrulline | Amino acid | 0.73 | ↓* |
| **105** | Taurine | Amino acid | 1.31 | ↑* |

Control group A v.s. EHLBS group * p<0.05

**Table S5 69 differential endogenous metabolites between the** **control group B and the BS group**

| **No.** | **Endogenous metabolites** | **Category** | **Content variance** | **trend** |
| --- | --- | --- | --- | --- |
| **1** | Glycocholic acid | Bile acid | 0.25 | ↓^*^ |
| **2** | Chenodeoxycholic acid glycine conjugate | Bile acid | 0.12 | ↓^*^ |
| **3** | Deoxycholic acid glycine conjugate | Bile acid | 0.36 | ↓^*^ |
| **4** | Lithocholic acid | Bile acid | 0.54 | ↓^*^ |
| **5** | Taurocholic acid | Bile acid | 2.11 | ↑^*^ |
| **6** | Hyodeoxycholtaurine | Bile acid | 2.07 | ↑^*^ |
| **7** | Tauroursodeoxycholic acid | Bile acid | 2.35 | ↑^*^ |
| **8** | PC(0:0/14:0) | Phospholipid (lysophosphatide) | 0.66 | ↓^*^ |
| **9** | PC(0:0/16:1(9Z)) | Phospholipid (lysophosphatide) | 0.63 | ↓^*^ |
| **10** | PC(0:0/17:0) | Phospholipid (lysophosphatide) | 0.71 | ↓^*^ |
| **11** | PC(0:0/18:1(9Z)) | Phospholipid (lysophosphatide) | 0.71 | ↓^*^ |
| **12** | PC(0:0/19:0) | Phospholipid (lysophosphatide) | 0.86 | ↓^*^ |
| **13** | PC(0:0/20:2(11Z,14Z)) | Phospholipid (lysophosphatide) | 0.49 | ↓^*^ |
| **14** | PC(0:0/20:3(8Z,11Z,14Z)) | Phospholipid (lysophosphatide) | 0.47 | ↓^*^ |
| **15** | PC(0:0/22:4(7Z,10Z,13Z,16Z)) | Phospholipid (lysophosphatide) | 0.78 | ↓^*^ |
| **16** | PC(14:0/0:0) | Phospholipid (lysophosphatide) | 0.67 | ↓^*^ |
| **17** | PC(16:1(9Z)/0:0) | Phospholipid (lysophosphatide) | 0.63 | ↓^*^ |
| **18** | PC(17:0/0:0) | Phospholipid (lysophosphatide) | 0.78 | ↓^*^ |
| **19** | PC(17:1(9Z)/0:0) | Phospholipid (lysophosphatide) | 0.53 | ↓^*^ |
| **20** | PC(18:0/0:0) | Phospholipid (lysophosphatide) | 0.98 | ↓^*^ |
| **21** | PC(18:1(9Z)/0:0) | Phospholipid (lysophosphatide) | 0.70 | ↓^*^ |
| **22** | PC(19:0/0:0) | Phospholipid (lysophosphatide) | 0.76 | ↓^*^ |
| **23** | PC(20:0/0:0) | Phospholipid (lysophosphatide) | 0.82 | ↓^*^ |
| **24** | PC(20:2(11Z,14Z)/0:0) | Phospholipid (lysophosphatide) | 0.50 | ↓^*^ |
| **25** | PC(20:3(8Z,11Z,14Z)/0:0) | Phospholipid (lysophosphatide) | 0.49 | ↓^*^ |
| **26** | PC(22:0/0:0) | Phospholipid (lysophosphatide) | 0.86 | ↓^*^ |
| **27** | PC(22:4(7Z,10Z,13Z,16Z)/0:0) | Phospholipid (lysophosphatide) | 0.76 | ↓^*^ |
| **28** | PC(24:0/0:0) | Phospholipid (lysophosphatide) | 0.82 | ↓^*^ |
| **29** | PC(0:0/18:0) | Phospholipid (lysophosphatide) | 1.14 | ↑^*^ |
| **30** | PC(O-16:0/0:0) | Phospholipid (lysophosphatide) | 1.14 | ↑* |
| **31** | PC(P-16:0/0:0) | Phospholipid (lysophosphatide) | 1.03 | ↑* |
| **32** | PC(P-18:0/0:0) | Phospholipid (lysophosphatide) | 1.25 | ↑* |
| **33** | PE(0:0/16:0) | Phospholipid (lysophosphatide) | 1.29 | ↑* |
| **34** | PE(0:0/18:0) | Phospholipid (lysophosphatide) | 1.50 | ↑* |
| **35** | PE(0:0/18:2(9Z,12Z)) | Phospholipid (lysophosphatide) | 1.42 | ↑* |
| **36** | PE(16:0/0:0) | Phospholipid (lysophosphatide) | 1.31 | ↑* |
| **37** | PE(18:0/0:0) | Phospholipid (lysophosphatide) | 1.52 | ↑* |
| **38** | PE(18:2(9Z,12Z)/0:0) | Phospholipid (lysophosphatide) | 1.39 | ↑* |
| **39** | PC(18:0/16:0) | Phospholipid (phosphatidylcholine) | 1.54 | ↑* |
| **40** | PC(18:0/18:0) | Phospholipid (phosphatidylcholine) | 1.29 | ↑* |
| **41** | PC(18:0/18:2(9Z,12Z)) | Phospholipid (phosphatidylcholine) | 1.18 | ↑* |
| **42** | PC(18:0/20:3(8Z,11Z,14Z)) | Phospholipid (phosphatidylcholine) | 0.63 | ↓* |
| **43** | PC(18:0/22:5(7Z,10Z,13Z,16Z,19Z)) | Phospholipid (phosphatidylcholine) | 0.77 | ↓* |
| **44** | PC(22:6(4Z,7Z,10Z,13Z,16Z,19Z)/14:0) | Phospholipid (phosphatidylcholine) | 0.57 | ↓* |
| **45** | PC(22:6(4Z,7Z,10Z,13Z,16Z,19Z)/18:0) | Phospholipid (phosphatidylcholine) | 1.36 | ↑* |
| **46** | PC(20:4(5Z,8Z,11Z,14Z)/14:0) | Phospholipid (phosphatidylcholine) | 0.44 | ↓* |
| **47** | PC(20:4(5Z,8Z,11Z,14Z)/15:0) | Phospholipid (phosphatidylcholine) | 0.57 | ↓* |
| **48** | PC(20:4(5Z,8Z,11Z,14Z)/17:0) | Phospholipid (phosphatidylcholine) | 0.69 | ↓* |
| **49** | PC(20:4(5Z,8Z,11Z,14Z)/18:1(9Z)) | Phospholipid (phosphatidylcholine) | 0.60 | ↓* |
| **50** | PC(20:4(5Z,8Z,11Z,14Z)/20:3(8Z,11Z,14Z)) | Phospholipid (phosphatidylcholine) | 0.68 | ↓* |
| **51** | PC(16:0/16:0) | Phospholipid (phosphatidylcholine) | 1.22 | ↑* |
| **52** | PC(O-16:0/16:0) | Phospholipid (plasmalogen) | 1.56 | ↑* |
| **53** | PE(22:6(4Z,7Z,10Z,13Z,16Z,19Z)/16:0) | Phospholipid (phosphatidylethanolamine) | 2.29 | ↑* |
| **54** | SM (d18:1/16:0) | Phospholipid (sphingomyelin) | 1.34 | ↑* |
| **55** | SM (d18:1/18:1(9Z)) | Phospholipid (sphingomyelin) | 1.43 | ↑* |
| **56** | SM(d16:1/16:0) | Phospholipid (sphingomyelin) | 1.28 | ↑* |
| **57** | SM(d16:1/18:0) | Phospholipid (sphingomyelin) | 1.34 | ↑* |
| **58** | SM(d16:1/18:1) | Phospholipid (sphingomyelin) | 1.33 | ↑* |
| **59** | SM(d16:1/20:1) | Phospholipid (sphingomyelin) | 1.43 | ↑* |
| **60** | SM(d18:0/16:0) | Phospholipid (sphingomyelin) | 1.94 | ↑* |
| **61** | SM(d18:1/18:0) | Phospholipid (sphingomyelin) | 2.37 | ↑* |
| **62** | SM(d18:2/24:0) | Phospholipid (sphingomyelin) | 1.35 | ↑* |
| **63** | SM(d18:2/24:1) | Phospholipid (sphingomyelin) | 1.36 | ↑* |
| **64** | Palmitoylcarnitine | Fatty acid | 1.57 | ↑* |
| **65** | Stearoylcarnitine | Fatty acid | 1.74 | ↑* |
| **66** | Glycine | Amino acid | 1.04 | ↓* |
| **67** | Valine | Amino acid | 1.13 | ↑* |
| **68** | Histidine | Amino acid | 1.16 | ↑* |
| **69** | Taurine | Amino acid | 1.08 | ↑* |

Control group B v.s. BS group *p<0.05

**Table S6 69 differential endogenous metabolites between the PRR treated group A and the EHLBS group**

| **No.** | **Endogenous metabolites** | **Category** | **Content variance** | **Trend** |
| --- | --- | --- | --- | --- |
| **1** | Tauro-*α*-muricholic acid | Bile acid | 2.54 | ↑* |
| **2** | Tauro-*β*-muricholic acid | Bile acid | 2.15 | ↑* |
| **3** | Taurocholic acid | Bile acid | 3.38 | ↑* |
| **4** | Taurodeoxycholic acid | Bile acid | 77.88 | ↑* |
| **5** | Hyodeoxycholtaurine acid | Bile acid | 2.80 | ↑* |
| **6** | Lithocholyltaurine acid | Bile acid | 1.60 | ↑* |
| **7** | Glycocholic acid | Bile acid | 4.07 | ↑* |
| **8** | Chenodeoxycholic acid glycine conjugate | Bile acid | 4.14 | ↑* |
| **9** | Deoxycholic acid glycine conjugate | Bile acid | 2.93 | ↑* |
| **10** | PC(16:0/18:1(9Z)) | Phospholipid (phosphatidylcholine) | 1.19 | ↑* |
| **11** | PC(16:0/20:4(5Z,8Z,11Z,14Z)) | Phospholipid (phosphatidylcholine) | 1.21 | ↑* |
| **12** | PC(18:2(9Z,12Z)/16:0) | Phospholipid (phosphatidylcholine) | 1.17 | ↑* |
| **13** | PC(18:2(9Z,12Z)/17:0) | Phospholipid (phosphatidylcholine) | 1.26 | ↑* |
| **14** | PC(20:4(5Z,8Z,11Z,14Z)/16:1(9Z)) | Phospholipid (phosphatidylcholine) | 1.25 | ↑* |
| **15** | PC(20:4(5Z,8Z,11Z,14Z)/18:0) | Phospholipid (phosphatidylcholine) | 1.17 | ↑* |
| **16** | PC(20:4(5Z,8Z,11Z,14Z)/14:0) | Phospholipid (phosphatidylcholine) | 0.81 | ↓* |
| **17** | PC(20:4(5Z,8Z,11Z,14Z)/18:2(9Z,12Z)) | Phospholipid (phosphatidylcholine) | 0.83 | ↓* |
| **18** | PC(22:6(4Z,7Z,10Z,13Z,16Z,19Z)/16:0) | Phospholipid (phosphatidylcholine) | 0.83 | ↓* |
| **19** | PC(22:6(4Z,7Z,10Z,13Z,16Z,19Z)/18:0) | Phospholipid (phosphatidylcholine) | 0.72 | ↓* |
| **20** | PC(14:0/18:2(9Z,12Z)) | Phospholipid (phosphatidylcholine) | 1.04 | ↑ |
| **21** | PC(18:0/18:2(9Z,12Z)) | Phospholipid (phosphatidylcholine) | 1.10 | ↑ |
| **22** | PC(18:2(9Z,12Z)/15:0) | Phospholipid (phosphatidylcholine) | 1.17 | ↑ |
| **23** | PC(O-16:0/16:1(9Z)) | Phospholipid (plasmalogen) | 1.65 | ↑* |
| **24** | PC(O-16:0/20:4(5Z,8Z,11Z,14Z)) | Phospholipid (plasmalogen) | 1.38 | ↑* |
| **25** | PC(O-16:0/22:4(7Z,10Z,13Z,16Z)) | Phospholipid (plasmalogen) | 1.50 | ↑* |
| **26** | PC(O-18:0/20:4(5Z,8Z,11Z,14Z)) | Phospholipid (plasmalogen) | 1.58 | ↑* |
| **27** | PC(P-16:0/20:4(5Z,8Z,11Z,14Z)) | Phospholipid (plasmalogen) | 1.53 | ↑* |
| **28** | PC(P-18:0/20:4(5Z,8Z,11Z,14Z)) | Phospholipid (plasmalogen) | 1.32 | ↑* |
| **29** | PC(P-18:0/20:5(5Z,8Z,11Z,14Z,17Z)) | Phospholipid (plasmalogen) | 1.40 | ↑* |
| **30** | PC(20:2(11Z,14Z)/0:0) | Phospholipid (lysophosphatide) | 0.67 | ↓* |
| **31** | PC(0:0/20:2(11Z,14Z)) | Phospholipid (lysophosphatide) | 0.65 | ↓* |
| **32** | PC(18:1(9Z)/0:0) | Phospholipid (lysophosphatide) | 0.77 | ↓* |
| **33** | PC(0:0/18:1(9Z)) | Phospholipid (lysophosphatide) | 0.78 | ↓* |
| **34** | PC(0:0/16:1(9Z)) | Phospholipid (lysophosphatide) | 0.66 | ↓* |
| **35** | PC(22:0/0:0) | Phospholipid (lysophosphatide) | 1.15 | ↑* |
| **36** | PC(P-18:0/0:0) | Phospholipid (lysophosphatide) | 1.27 | ↑* |
| **37** | PC(P-16:0/0:0) | Phospholipid (lysophosphatide) | 1.24 | ↑* |
| **38** | PC(O-18:0/0:0) | Phospholipid (lysophosphatide) | 1.18 | ↑* |
| **39** | PE(18:1(9Z)/0:0) | Phospholipid (lysophosphatide) | 0.84 | ↓* |
| **40** | PE(22:4(7Z,10Z,13Z,16Z)/0:0) | Phospholipid (lysophosphatide) | 0.78 | ↓* |
| **41** | (3'-sulfo)Galbeta-Cer(d18:1/16:0(2OH)) | Phospholipid (ceramide) | 0.96 | ↓* |
| **42** | Cer(d18:0/14:0) | Phospholipid (ceramide) | 0.74 | ↓* |
| **43** | Etn-1-P-Cer(d14:1/18:0) | Phospholipid (ceramide) | 1.28 | ↑* |
| **44** | SM (d18:1/16:0) | Phospholipid (sphingomyelin) | 1.25 | ↑* |
| **45** | SM (d18:1/18:1(9Z)) | Phospholipid (sphingomyelin) | 1.35 | ↑* |
| **46** | SM(d16:0/20:0) | Phospholipid (sphingomyelin) | 3.07 | ↑* |
| **47** | SM(d16:1/17:0) | Phospholipid (sphingomyelin) | 1.28 | ↑* |
| **48** | SM(d16:1/18:0) | Phospholipid (sphingomyelin) | 1.25 | ↑* |
| **49** | SM(d16:1/18:1) | Phospholipid (sphingomyelin) | 1.21 | ↑* |
| **50** | SM(d16:1/20:1) | Phospholipid (sphingomyelin) | 1.35 | ↑* |
| **51** | SM(d16:1/24:1) | Phospholipid (sphingomyelin) | 1.14 | ↑* |
| **52** | SM(d18:0/16:0) | Phospholipid (sphingomyelin) | 1.77 | ↑* |
| **53** | SM(d18:1/18:0) | Phospholipid (sphingomyelin) | 1.47 | ↑* |
| **54** | SM(d18:1/20:0) | Phospholipid (sphingomyelin) | 1.34 | ↑* |
| **55** | SM(d18:2/18:1) | Phospholipid (sphingomyelin) | 1.66 | ↑* |
| **56** | SM(d18:2/20:0) | Phospholipid (sphingomyelin) | 1.39 | ↑* |
| **57** | SM(d18:2/24:1) | Phospholipid (sphingomyelin) | 1.15 | ↑* |
| **58** | Alanine | Amino acid | 1.32 | ↑* |
| **59** | Arginine | Amino acid | 1.14 | ↑* |
| **60** | Glutamic acid | Amino acid | 1.24 | ↑* |
| **61** | Glycine | Amino acid | 1.05 | ↑ |
| **62** | Histidine | Amino acid | 1.11 | ↑* |
| **63** | Lysine | Amino acid | 1.06 | ↑* |
| **64** | Serine | Amino acid | 1.18 | ↑ |
| **65** | Phenylalanine | Amino acid | 1.19 | ↑* |
| **66** | Threonine | Amino acid | 1.06 | ↑ |
| **67** | Ornithine | Amino acid | 1.15 | ↑* |
| **68** | Taurine | Amino acid | 2.54 | ↑* |
| **69** | Citrulline | Amino acid | 1.11 | ↑* |

PRR-treated group A v.s. EHLBS group *p<0.05

**Table S7 54 differential endogenous metabolites between the PRR treated group B and the BS group**

| **No.** | **Endogenous metabolites** | **Category** | **Content variance** | **Trend** |
| --- | --- | --- | --- | --- |
| **1** | Cholalic acid | Bile acid | 0.86 | ↓^*^ |
| **2** | *β*-muricholic acid | Bile acid | 0.54 | ↓^*^ |
| **3** | Glycoursodeoxycholic acid | Bile acid | 1.56 | ↑^*^ |
| **4** | Chenodeoxycholic acid glycine conjugate | Bile acid | 1.56 | ↑^*^ |
| **5** | Tauro-*α*-muricholic acid | Bile acid | 1.34 | ↑^*^ |
| **6** | Tauro-*β*-muricholic acid | Bile acid | 1.34 | ↑^*^ |
| **7** | Glycocholic acid | Bile acid | 2.84 | ↑^*^ |
| **8** | Deoxycholic acid glycine conjugate | Bile acid | 3.09 | ↑^*^ |
| **9** | Hyodeoxycholtaurine | Bile acid | 1.59 | ↑^*^ |
| **10** | Lithocholyltaurine | Bile acid | 5.18 | ↑^*^ |
| **11** | Tauroursodeoxycholic acid | Bile acid | 1.72 | ↑^*^ |
| **12** | PC(22:6(4Z,7Z,10Z,13Z,16Z,19Z)/0:0) | Phospholipid (lysophosphatide) | 0.72 | ↓^*^ |
| **13** | PC(0:0/18:0) | Phospholipid (lysophosphatide) | 0.84 | ↓^*^ |
| **14** | PC(0:0/18:2(9Z,12Z)) | Phospholipid (lysophosphatide) | 0.76 | ↓^*^ |
| **15** | PC(0:0/20:3(8Z,11Z,14Z)) | Phospholipid (lysophosphatide) | 0.62 | ↓^*^ |
| **16** | PC(0:0/20:4(5Z,8Z,11Z,14Z)) | Phospholipid (lysophosphatide) | 0.82 | ↓^*^ |
| **17** | PC(0:0/20:5(5Z,8Z,11Z,14Z,17Z)) | Phospholipid (lysophosphatide) | 0.74 | ↓^*^ |
| **18** | PC(0:0/22:4(7Z,10Z,13Z,16Z)) | Phospholipid (lysophosphatide) | 0.84 | ↓^*^ |
| **19** | PC(0:0/22:6(4Z,7Z,10Z,13Z,16Z,19Z)) | Phospholipid (lysophosphatide) | 0.70 | ↓^*^ |
| **20** | PC(18:0/0:0) | Phospholipid (lysophosphatide) | 0.97 | ↓^*^ |
| **21** | PC(18:1(9Z)/0:0) | Phospholipid (lysophosphatide) | 0.87 | ↓^*^ |
| **22** | PC(18:2(9Z,12Z)/0:0) | Phospholipid (lysophosphatide) | 0.79 | ↓^*^ |
| **23** | PC(20:3(8Z,11Z,14Z)/0:0) | Phospholipid (lysophosphatide) | 0.66 | ↓^*^ |
| **24** | PC(20:4(5Z,8Z,11Z,14Z)/0:0) | Phospholipid (lysophosphatide) | 0.86 | ↓^*^ |
| **25** | PC(20:5(5Z,8Z,11Z,14Z,17Z)/0:0) | Phospholipid (lysophosphatide) | 0.73 | ↓^*^ |
| **26** | PC(22:0/0:0) | Phospholipid (lysophosphatide) | 0.78 | ↓^*^ |
| **27** | PC(22:4(7Z,10Z,13Z,16Z)/0:0) | Phospholipid (lysophosphatide) | 0.85 | ↓^*^ |
| **28** | PE(0:0/18:0) | Phospholipid (lysophosphatide) | 0.84 | ↓^*^ |
| **29** | PE(18:0/0:0) | Phospholipid (lysophosphatide) | 0.88 | ↓^*^ |
| **30** | PE(18:1(9Z)/0:0) | Phospholipid (lysophosphatide) | 0.83 | ↓^*^ |
| **31** | PE(P-18:0/0:0) | Phospholipid (lysophosphatide) | 0.67 | ↓^*^ |
| **32** | PC(16:0/16:0) | Phospholipid (phosphatidylcholine) | 0.93 | ↓^*^ |
| **33** | PC(16:0/20:4(5Z,8Z,11Z,14Z)) | Phospholipid (phosphatidylcholine) | 0.99 | ↓^*^ |
| **34** | PC(18:0/16:0) | Phospholipid (phosphatidylcholine) | 0.78 | ↓^*^ |
| **35** | PC(18:0/18:2(9Z,12Z)) | Phospholipid (phosphatidylcholine) | 0.84 | ↓^*^ |
| **36** | PC(18:0/20:3(8Z,11Z,14Z)) | Phospholipid (phosphatidylcholine) | 0.67 | ↓^*^ |
| **37** | PC(18:2(9Z,12Z)/15:0) | Phospholipid (phosphatidylcholine) | 0.76 | ↓^*^ |
| **38** | PC(18:2(9Z,12Z)/17:0) | Phospholipid (phosphatidylcholine) | 0.80 | ↓^*^ |
| **39** | PC(20:4(5Z,8Z,11Z,14Z)/15:0) | Phospholipid (phosphatidylcholine) | 0.71 | ↓^*^ |
| **40** | PC(20:4(5Z,8Z,11Z,14Z)/16:1(9Z)) | Phospholipid (phosphatidylcholine) | 0.50 | ↓^*^ |
| **41** | PC(20:4(5Z,8Z,11Z,14Z)/17:0) | Phospholipid (phosphatidylcholine) | 0.74 | ↓^*^ |
| **42** | PC(20:4(5Z,8Z,11Z,14Z)/18:0) | Phospholipid (phosphatidylcholine) | 0.80 | ↓^*^ |
| **43** | PC(22:6(4Z,7Z,10Z,13Z,16Z,19Z)/16:0) | Phospholipid (phosphatidylcholine) | 0.85 | ↓* |
| **44** | PC(22:6(4Z,7Z,10Z,13Z,16Z,19Z)/18:0) | Phospholipid (phosphatidylcholine) | 0.82 | ↓* |
| **45** | PC(16:0/14:0) | Phospholipid (phosphatidylcholine) | 0.82 | ↓* |
| **46** | PC(20:4(5Z,8Z,11Z,14Z)/18:2(9Z,12Z)) | Phospholipid (phosphatidylcholine) | 0.77 | ↓* |
| **47** | PC(20:4(5Z,8Z,11Z,14Z)/20:3(8Z,11Z,14Z)) | Phospholipid (phosphatidylcholine) | 0.82 | ↓* |
| **48** | PC(20:4(5Z,8Z,11Z,14Z)/20:4(5Z,8Z,11Z,14Z)) | Phospholipid (phosphatidylcholine) | 0.78 | ↓* |
| **49** | PC(O-16:0/20:4(5Z,8Z,11Z,14Z)) | Phospholipid (plasmalogen) | 0.76 | ↓* |
| **50** | SM(d16:1/16:0) | Phospholipid (sphingomyelin) | 0.78 | ↓* |
| **51** | SM(d18:2/24:0) | Phospholipid (sphingomyelin) | 0.81 | ↓* |
| **52** | Valine | Amino acid | 1.16 | ↑* |
| **53** | Taurine | Amino acid | 1.13 | ↑* |
| **54** | Cer(d18:1/24:0) | Ceramide | 1.84 | ↑* |

PRR-treated group B v.s. BS group *p<0.05

**Table S8 Pathways regulated by PRR in the EHLBS stage**

| **Pathway Name** | **Total** | **Holm adjust p** | **Impact** |
| --- | --- | --- | --- |
| Aminoacyl-tRNA biosynthesis | 67 | 0.00 | 0.14 |
| Cyanoamino acid metabolism | 6 | 0.07 | 0.00 |
| Nitrogen metabolism | 9 | 0.15 | 0.00 |
| Methane metabolism | 9 | 0.15 | 0.40 |
| Arginine and proline metabolism | 44 | 0.31 | 0.24 |
| Glutathione metabolism | 26 | 1.00 | 0.01 |
| Glycine, serine and threonine metabolism | 32 | 1.00 | 0.53 |
| Linoleic acid metabolism | 5 | 1.00 | 0.00 |
| Biotin metabolism | 5 | 1.00 | 0.00 |
| alpha-Linolenic acid metabolism | 9 | 1.00 | 0.00 |
| Ether lipid metabolism | 13 | 1.00 | 0.21 |
| Selenoamino acid metabolism | 15 | 1.00 | 0.00 |
| Histidine metabolism | 15 | 1.00 | 0.24 |
| Sphingolipid metabolism | 21 | 1.00 | 0.00 |
| Alanine, aspartate and glutamate metabolism | 24 | 1.00 | 0.00 |
| Porphyrin and chlorophyll metabolism | 27 | 1.00 | 0.00 |
| Cysteine and methionine metabolism | 28 | 1.00 | 0.02 |
| Glycerophospholipid metabolism | 30 | 1.00 | 0.14 |
| Arachidonic acid metabolism | 36 | 1.00 | 0.00 |
| Primary bile acid biosynthesis | 46 | 1.00 | 0.03 |

Total is the total number in the pathway; the Holm adjust p is the p value adjusted by Holm-Bonferroni method; the impact is the pathway impact value calculated from pathway topology analysis.

**Table S9 Pathways regulated by PRR in the BS stage**

| **Pathway name** | **Total** | **Holm adjust p** | **Impact** |
| --- | --- | --- | --- |
| Primary bile acid biosynthesis | 46 | 0.49 | 0.06 |
| Linoleic acid metabolism | 5 | 1.00 | 0.00 |
| alpha-Linolenic acid metabolism | 9 | 1.00 | 0.00 |
| Glycerophospholipid metabolism | 30 | 1.00 | 0.14 |
| Arachidonic acid metabolism | 36 | 1.00 | 0.00 |

Total is the total number in the pathway; the Holm adjust p is the p value adjusted by Holm-Bonferroni method; the impact is the pathway impact value calculated from pathway topology analysis.

Supplementary Information 4

Identification of the original constituents and metabolites of PRR

# Analysis method of the original constituents and metabolites of PRR

The analysis of the original constituents and metabolites of PRR was performed using a Dionex UltiMate 3000 LC system (Thermo Fisher Scientific, Massachusetts, USA) connected to a Q Exactive mass spectrometer (Thermo Fisher Scientific, Massachusetts, USA). Separation was performed on a Waters HSS T3 column (2.1 mm × 100 mm, 1.8 μm) kept at 50 ℃, and the flow rate was maintained at 0.3 mL/min. The gradient mobile phase consisted of a mixture of 0.1% formic acid in water (A) and acetonitrile (B). The gradient elution was performed as follows: 0–1.5 min, 0% B; 1.5–6.5 min, 0%–20% B; 6.5–10 min, 20%–40% B; 10–11 min, 40%–100% B; 11.1–12.5 min, 100%–0% B. The sample solution (3 µL) was injected for each run. During the entire analysis, all the samples were maintained at 4°C. The spray voltage was 3700 V, evaporation temperature was 350°C, sheath gas was 28 arb., auxiliary gas was 8 arb., capillary temperature was 320°C, and S-lens RF was 50. The scan was in negative mode and the scan range was *m/z* 100–1500.

# Results

The identified original constituents and metabolites of PRR were listed in the Tables S10 and S11.

**Table S10 Information of 8 original constituents of PRR**

| **No.** | **t_R_(min)** | **Formula** | **Meas.**  **(Da)** | **Pred.**  **(Da)** | **Error**  **(ppm)** | **Major fragment ions detected in negative ion mode** | **Identification result** |
| --- | --- | --- | --- | --- | --- | --- | --- |
| **A1** | 4.45 | C_16_H_24_O_9_ | 405.1395 | 405.1402 | -0.86 | 405.1495, 359.1322, 197.0877, 179.0814 | Glucopyranosyl-paeonisuffrone |
| **A2** | 4.66 | C_16_H_24_O_8_ | 389.1450 | 389.1453 | -0.06 | 389.1450, 343.1408, 182.0843, 181.0963, 159.0346, 151.0810 | Mudanpioside F |
| **A3** | 4.58 | C_16_H_24_O_10_ | 375.1295 | 375.1297 | -0.47 | 375.1295, 345.1177, 195.0670, 183.0674, 139.0921 | Desbenzoylpaeoniflorin |
| **A4** | 4.41 | C_16_H_24_O_10_ | 375.1295 | 375.1297 | -0.47 | 375.1295, 345.1215, 195.0729, 183.0691, 151.0823 | Desbenzoylpaeoniflorin isomer II |
| **A5** | 5.12 | C_23_H_28_O_11_ | 479.1549 | 479.1559 | -1.23 | 479.1549, 449.1436, 327.1055, 165.0571 | Paeoniflorin |
| **A6** | 4.76 | C_23_H_28_O_12_ | 495.1505 | 495.1508 | -0.61 | 495.1505, 465.1402, 299.0801, 281.0645, 367.0878, 239.0575, 165.0612 | Oxypaeoniflorin |
| **A7** | 6.65 | C_16_H_10_O_8_ | 329.0299 | 329.0303 | -0.15 | 329.0299, 314.0060, 298.9813, 270.9871 | 3,7- or 3,8-dimethyl ellagic acid |
| **A8** | 4.54 | C_17_H_26_O_10_ | 435.1500 | 435.1508 | -0.52 | 435.1500, 389.1447, 227.0936, 195.0660, 165.0580, 149.0640 | 4-*O*-methyldesbenzoylpaeoniflorin |

**Table S11 Information of 23 metabolites of PRR**

| **No.** | **t_R_**  **(min)** | **Formula** | **Meas.**  **(Da)** | **Pred.**  **(Da)** | **Error**  **(ppm)** | **Identification result** | **Possible original compound** | **Major fragment ions detected in negative ion mode** | **THBSS stage** |
| --- | --- | --- | --- | --- | --- | --- | --- | --- | --- |
| **M1** | 4.90 | C_22_H_24_O_12_ | 479.1191 | 479.1195 | -2.03 | 3′-O-methyl (epi) catechin 5*-O*-glucuronide | (epi)catechin-related | 479.1191, 303.0872, 175.0237, 137.0228, 113.0229, 85.0280 | EHLBS, BS |
| **M2** | 5.02 | C_9_H_10_O_6_S | 245.0122 | 245.0125 | -1.22 | 3-hydroxy phenylpropionic acid sulfate | (epi)catechin-related | 245.0121, 165.0546, 121.0645 | EHLBS, BS |
| **M3** | 4.88 | C_10_H_12_O_7_S | 275.0228 | 275.0231 | -1.35 | 3-hydroxy-4-methoxy-phenylpropionic acid sulfate | (epi)catechin-related | 275.0228, 195.0652, 177.0550, 155.0085, 121.0029, 112.0026 | EHLBS, BS |
| **M4** | 4.96 | C_10_H_10_O_7_S | 273.0071 | 273.0074 | -0.43 | Ferulic acid sulfate | (epi)catechin-related | 273.0071, 193.0498, 134.0359, 178.0261, 149.0595 | EHLBS |
| **M5** | 5.45 | C_8_H_8_O_6_S | 230.9958 | 230.9969 | -4.93 | 3-hydroxy phenylacetic acid sulfate | (epi)catechin-related | 230.9958, 151.0388, 135.0106, 74.0232 | EHLBS, BS |
| **M6** | 4.76 | C_7_H_6_O_6_S | 216.9795 | 216.9812 | -8.16 | 3- or 4-hydroxy benzoic acid sulfate | (epi)catechin related  gallic acid-related | 216.9795, 137.0231, 93.0330 | EHLBS |
| **M7** | 4.61 | C_8_H_8_O_8_S | 262.9861 | 262.9867 | -2.51 | 4-*O*-methylgallic acid sulfate | Gallic acid-related | 262.9861, 183.0288, 168.0052, 124.0151 | EHLBS |
| **M8** | 5.00 | C_9_H_10_O_5_ | 277.0022 | 277.0024 | -0.91 | 3,4-di-*O*-methyl gallic acid sulfate | Gallic acid-related | 277.0022, 197.0446, 182.0211, 138.0311, 123.0073, 75.0073 | EHLBS |
| **M9** | 4.91 | C_12_H_14_O_7_ | 269.0667 | 269.0667 | 0.00 | Phenol glucuronide | Gallic acid-related | 269.0667, 175.0236, 113.0229, 93.0330, 85.0279, 75.0072, 59.0123 | EHLBS, BS |
| **M10** | 4.97 | C_16_H_26_O_8_ | 345.1555 | 345.1555 | 0.00 | C_10_H_18_O_2_ glucuronide | Paeoniflorin-related | 345.1555, 327.1459, 300.0041, 175.0243, 157.0126, 113.0229, 129.0108 | EHLBS |
| **M11** | 5.23 | C_16_H_26_O_8_ | 345.1554 | 345.1555 | -0.80 | C_10_H_18_O_2_ glucuronide | Paeoniflorin-related | 345.1555, 327.1459, 300.0041, 175.0243,157.0126, 113.0229, 129.0108 | EHLBS, BS |
| **M12** | 5.36 | C_16_H_26_O_8_ | 345.1554 | 345.1555 | -0.80 | C_10_H_18_O_2_ glucuronide | Paeoniflorin-related | 345.1555, 327.1459, 300.0041, 175.0243, 157.0126, 113.0229, 129.0108 | EHLBS |
| **M13** | 5.60 | C_16_H_26_O_8_ | 345.1554 | 345.1555 | -0.80 | C_10_H_18_O_2_ glucuronide | Paeoniflorin-related | 345.1555, 327.1459, 300.0041, 175.0243, 157.0126, 113.0229, 129.0108 | EHLBS, BS |
| **M14** | 5.72 | C_16_H_26_O_8_ | 345.1555 | 345.1555 | 0.00 | C_10_H_18_O_2_ glucuronide | Paeoniflorin-related | 345.1555, 327.1459, 300.0041, 175.0243, 157.0126, 113.0229, 129.0108 | EHLBS |
| **M15** | 5.79 | C_16_H_26_O_8_ | 345.1554 | 345.1555 | -0.00 | C_10_H_18_O_2_ glucuronide | Paeoniflorin-related | 345.1555, 327.1459, 300.0041, 175.0243, 157.0126, 113.0229, 129.0108 | EHLBS, BS |
| **M16** | 5.92 | C_16_H_26_O_8_ | 345.1555 | 345.1555 | -0.00 | C_10_H_18_O_2_ glucuronide | Paeoniflorin-related | 345.1555, 327.1459, 300.0041, 175.0243, 157.0126, 113.0229, 129.0108 | EHLBS, BS |
| **M17** | 4.45 | C_16_H_26_O_10_ | 377.1450 | 377.1453 | -1.50 | C_10_H_18_O_4_ glucuronide | Paeoniflorin-related | 377.1450, 359.1347, 174.9550, 193.0344, 113.0228, 85.0278, 75.0071 | EHLBS |
| **M18** | 5.34 | C_16_H_22_O_9_ | 357.1190 | 357.1191 | -1.08 | C_10_H_14_O_3_ glucuronide | Paeoniflorin-related | 357.1190, 174.9549, 113.0230, 85.0279 | EHLBS, BS |
| **M19** | 4.86 | C_10_H_12_O_7_S | 275.0224 | 275.0231 | -2.05 | 3-methoxy-4-hydroxy-phenylpropionic acid sulfate | (epi)catechin-related | 275.0224, 195.0650, 177.0547, 121.0029 | EHLBS, BS |
| **M20** | 4.62 | C_14_H_16_O_9_ | 327.0719 | 327.0722 | -0.78 | C_8_H_8_O_3_ glucuronide | Paeoniflorin-related | 327.0719, 283.0826, 175.0246, 151.0399, 113.0242 | EHLBS, BS |
| **M21** | 5.03 | C_14_H_16_O_9_ | 327.0728 | 327.0722 | 1.97 | C_8_H_8_O_3_ glucuronide | Paeoniflorin-related | 327.0728, 283.0829, 175.0246, 151.0400, 113.0243, 107.0500 | EHLBS, BS |
| **M22** | 5.77 | C_13_H_14_O_8_ | 297.0979 | 297.0982 | 0.00 | Benzoyl glucuronide | (epi)catechin-related | 297.0978, 279.0853, 175.0237, 157.0128, 121.0644, 113.0229, 85.0279 | EHLBS, BS |
| **M23** | 6.49 | C_16_H_26_O_8_ | 345.1555 | 345.1555 | -0.00 | C_10_H_18_O_2_ glucuronide | Paeoniflorin-related | 345.1555, 327.1459, 300.0041, 175.0243, 157.0126, 113.0229, 129.0108 | BS |


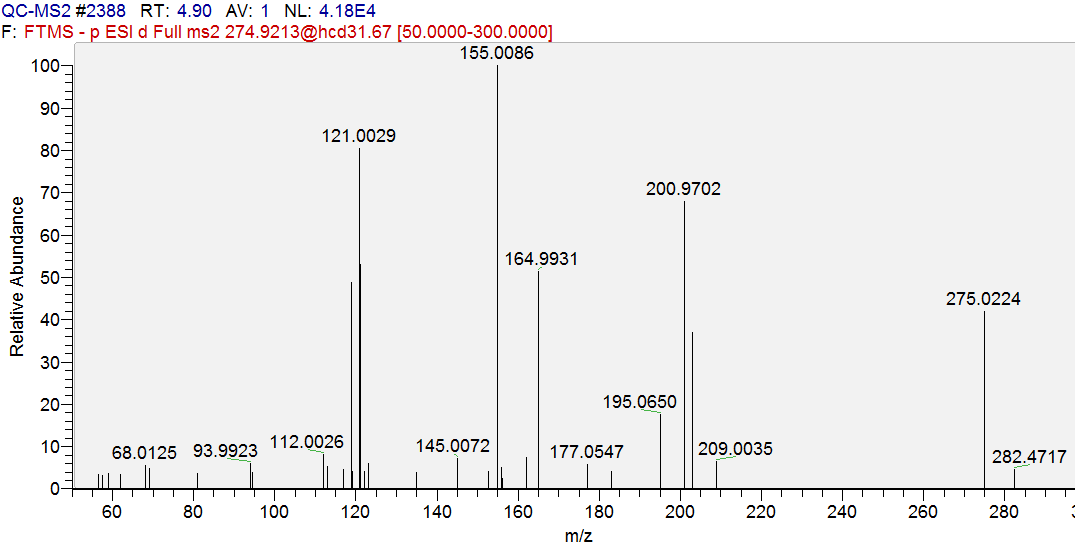


**Fig. S2 Product ion spectra of metabolite M3 in negative mode**


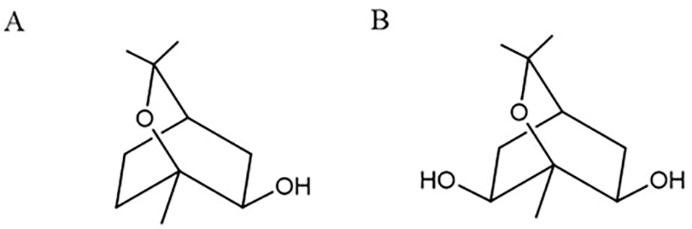


**Fig. S3 Structural formula of (A) 2-hydroxyl-1,8-cineole, and (B) epomediol. The aglycones of M11, M13, M15, and M16 are a series of isomers formed due to different positions of hydroxyl group.**

Supplementary Information 5

Screening of potential effective substances of PRR

**Table S12 The established PLS regression equations**

| **THBSS stages** | **PLS regression equations** | **R^2^X** | **Q^2^** |
| --- | --- | --- | --- |
| **EHLBS stage** | Y(IL-6)=-0.10M15+0.21M4-0.18M21-0.08M20-0.09M14-0.11M11+0.012M2-0.19M17-0.099M12-0.13M18-0.20M6-0.047M16-0.19M3-0.099A4-0.099M13-0.047M10+0.054A1+0.04M1+0.042A2+0.048A5+0.038A8+0.007M8+0.0073A6-0.055M9-0.095M22-0.014M19-0.059A7-0.10M5-0.062A3+0.079M7 | 0.669 | 0.720 |
|  | Y(TNF-α)=0.21M20-0.17A7-0.12A5-0.12A6+0.15M16+0.11M12+0.09M15-0.04M19+0.13M8+0.13A3+0.076M14+0.091M4-0.091A1-0.019M11-0.055M7-0.084A2-0.043M2-0.069M3-0.089M13-0.089M13-0.067M10+0.059M5-0.016M9+0.061M17-0.057M1+0.011A8-0.058M21+0.047M6-0.020M22-0.017A4+0.015M18 | 0.694 | 0.541 |
| **BS stage** | Y(whole blood viscosity)=-0.43M22+0.29M5-0.18M2+0.3M3  +0.27A8-0.15M1-0.097M16-0.13M20-0.12A6-0.081A7-0.045A5+0.11M19+0.15M1-0.11M18-0.017M21-0.024A2+0.17M9-0.0061M13-0.20M15+0.028M11+0.11M23 | 0.837 | 0.665 |
|  | Y(PT)=-0.30A7+0.22M23+0.16M18+0.14M15+0.27M1+0.12M11+0.078M13+0.068M16-0.018M21+0.025M19+0.029M20-0.095A4-0.059A5-0.065A6+0.15M2+0.14M9+0.012A8-0.050M3+0.057M5-0.0043M22 | 0.765 | 0.692 |

**Table S13 The regression coefficients and VIP values of independent variables (IL-6)**

| **Var ID (Primary)** | **VIP** | **M1.CoeffCS[4]**  **(Regression coefficient)** |
| --- | --- | --- |
| **M15** | 1.35 | -0.10 |
| **M4** | 1.33 | 0.21 |
| **M21** | 1.30 | -0.18 |
| **M20** | 1.21 | -0.08 |
| **M14** | 1.20 | -0.09 |
| **M11** | 1.20 | -0.11 |
| **M2** | 1.18 | 0.012 |
| **M17** | 1.16 | -0.19 |
| **M12** | 1.16 | -0.099 |
| **M18** | 1.15 | -0.13 |
| **M6** | 1.13 | -0.20 |
| **M16** | 1.13 | -0.047 |
| **M3** | 1.12 | -0.19 |
| **A4** | 1.11 | -0.099 |
| **M13** | 1.10 | -0.099 |
| **M10** | 0.90 | -0.047 |
| **A1** | 0.88 | 0.054 |
| **M1** | 0.87 | 0.040 |
| **A2** | 0.87 | 0.042 |
| **A5** | 0.85 | 0.048 |
| **A8** | 0.82 | 0.038 |
| **M8** | 0.80 | 0.007 |
| **A6** | 0.77 | 0.0073 |
| **M9** | 0.75 | -0.055 |
| **M22** | 0.72 | -0.095 |
| **M19** | 0.70 | -0.014 |
| **A7** | 0.63 | -0.059 |
| **M5** | 0.67 | -0.10 |
| **A3** | 0.61 | -0.062 |
| **M7** | 0.54 | 0.079 |

VIP＞1 indicated that the independent variable had a significant contribution to the dependent variable. Regression coefficient＜0 indicated a negative correlation between the independent variable and the dependent variable. Regression coefficient＞0 indicated a positive correlation between the independent variable and the dependent variable.

**Table S14 The regression coefficients and VIP values of independent variables (TNF-α)**

| **Var ID (Primary)** | **VIP** | **M1.CoeffCS[4]**  **(Regression coefficient)** |
| --- | --- | --- |
| **M20** | 2.08 | 0.21 |
| **A7** | 1.84 | -0.17 |
| **A5** | 1.61 | -0.12 |
| **A6** | 1.55 | -0.12 |
| **M16** | 1.51 | 0.15 |
| **M12** | 1.35 | 0.11 |
| **M15** | 1.24 | 0.09 |
| **M19** | 1.11 | -0.04 |
| **M8** | 1.05 | 0.13 |
| **A3** | 0.92 | 0.13 |
| **M14** | 0.90 | 0.076 |
| **M4** | 0.87 | 0.091 |
| **A1** | 0.87 | -0.091 |
| **M11** | 0.86 | -0.019 |
| **M7** | 0.84 | -0.055 |
| **A2** | 0.80 | -0.084 |
| **M2** | 0.79 | -0.043 |
| **M3** | 0.76 | -0.069 |
| **M13** | 0.72 | -0.089 |
| **M10** | 0.69 | -0.067 |
| **M5** | 0.59 | 0.059 |
| **M9** | 0.54 | -0.016 |
| **M17** | 0.50 | 0.061 |
| **M1** | 0.46 | -0.057 |
| **A8** | 0.45 | 0.011 |
| **M21** | 0.42 | -0.058 |
| **M6** | 0.41 | 0.047 |
| **M22** | 0.40 | -0.020 |
| **A4** | 0.20 | -0.017 |
| **M18** | 0.17 | 0.015 |

VIP＞1 indicated that the independent variable had a significant contribution to the dependent variable. Regression coefficient＜0 indicated a negative correlation between the independent variable and the dependent variable. Regression coefficient＞0 indicated a positive correlation between the independent variable and the dependent variable.

**Table S15 The regression coefficients and VIP values of independent variables (the whole blood viscosity)**

| **Var ID (Primary)** | **VIP** | **M1.CoeffCS[4]**  **(Regression coefficient)** |
| --- | --- | --- |
| **M22** | 1.94 | -0.43 |
| **M5** | 1.43 | 0.29 |
| **M2** | 1.40 | -0.18 |
| **M3** | 1.23 | 0.30 |
| **A8** | 1.09 | 0.27 |
| **M16** | 1.02 | -0.097 |
| **M20** | 0.98 | -0.13 |
| **A6** | 0.97 | -0.12 |
| **A7** | 0.97 | -0.081 |
| **A5** | 0.89 | -0.045 |
| **M19** | 0.82 | 0.11 |
| **M1** | 0.80 | 0.15 |
| **M18** | 0.78 | -0.11 |
| **M21** | 0.75 | -0.017 |
| **A2** | 0.69 | -0.24 |
| **M9** | 0.65 | 0.17 |
| **M13** | 0.64 | -0.0061 |
| **M15** | 0.60 | -0.20 |
| **M11** | 0.60 | 0.028 |
| **M23** | 0.58 | 0.11 |

VIP＞1 indicated that the independent variable had a significant contribution to the dependent variable. Regression coefficient＜0 indicated a negative correlation between the independent variable and the dependent variable. Regression coefficient＞0 indicated a positive correlation between the independent variable and the dependent variable.

**Table S16 The regression coefficients and VIP values of independent variables (PT)**

| **Var ID (Primary)** | **VIP** | **M1.CoeffCS[3]**  **(Regession coefficient)** |
| --- | --- | --- |
| **A7** | 1.63 | -0.30 |
| **M23** | 1.51 | 0.22 |
| **M18** | 1.31 | 0.16 |
| **M15** | 1.30 | 0.14 |
| **M1** | 1.23 | 0.27 |
| **M11** | 1.23 | 0.12 |
| **M13** | 1.06 | 0.078 |
| **M16** | 1.01 | 0.068 |
| **M21** | 0.98 | -0.018 |
| **M19** | 0.94 | 0.025 |
| **M20** | 0.90 | 0.029 |
| **A2** | 0.82 | -0.095 |
| **A5** | 0.80 | -0.059 |
| **A6** | 0.77 | -0.065 |
| **M2** | 0.69 | 0.15 |
| **M9** | 0.65 | 0.14 |
| **A8** | 0.65 | 0.012 |
| **M3** | 0.59 | -0.050 |
| **M5** | 0.39 | 0.057 |
| **M22** | 0.35 | -0.0043 |

VIP＞1 indicated that the independent variable had a significant contribution to the dependent variable. Regression coefficient＞0 indicated a positive correlation between the independent variable and the dependent variable. Regression coefficient＜0 indicated a negative correlation between the independent variable and the dependent variable.
